# Survey on Context-Aware Radio Frequency-Based Sensing

**DOI:** 10.3390/s25030602

**Published:** 2025-01-21

**Authors:** Eugene Casmin, Rodolfo Oliveira

**Affiliations:** 1 Departamento de Engenharia Electrotécnica e de Computadores, Faculdade de Ciências e Tecnologia (FCT), Universidade Nova de Lisboa, 2829-516 Caparica, Portugal; e.owilla@campus.fct.unl.pt; 2 Instituto de Telecomunicações, 1049-001 Lisbon, Portugal

**Keywords:** radio frequency spectrum sensing, context-aware real-time detection, machine learning-aided detection and classification

## Abstract

Radio frequency (RF) spectrum sensing is critical for applications requiring precise object and posture detection and classification. This survey aims to provide a focused review of context-aware RF-based sensing, emphasizing its principles, advancements, and challenges. It specifically examines state-of-the-art techniques such as phased array radar, synthetic aperture radar, and passive RF sensing, highlighting their methodologies, data input domains, and spatial diversity strategies. The paper evaluates feature extraction methods and machine learning approaches used for detection and classification, presenting their accuracy metrics across various applications. Additionally, it investigates the integration of RF sensing with other modalities, such as inertial sensors, to enhance context awareness and improve performance. Challenges like environmental interference, scalability, and regulatory constraints are addressed, with insights into real-world mitigation strategies. The survey concludes by identifying emerging trends, practical applications, and future directions for advancing RF sensing technologies.

## 1. Introduction

With the recent exponential growth of technology accompanied by corresponding demands for efficiency, sustainability, resilience, safety, security, and automation, RF-based sensing has established itself as an indispensable asset for object, posture, and motion detection and classification. Active RF-based systems, which emit controlled electromagnetic waves to probe the surrounding environment, alongside passive RF-based systems, which alternatively employ ambient electromagnetic signals for the same purpose, have experienced unforeseen advancements lately. Coupled with context awareness, which adds a dimension aimed at the understanding and interpretation of the implied situation of target entities in the sensing environment, the potential for use cases in various fields is enormous. This literature review survey delves into the vast body of works comprising the practical application of active and passive RF-based sensing for object motion and posture detection and classification, unveiling the wide range of methodologies, myriad of data input domains, DL classification techniques, and the accompanying evaluation metrics achieved across an array of useful quotidian applications. The studies considered are focused on presenting AI-driven, sustainable, and resilient human-centric solutions to problems faced in quotidian life.

Context-aware represents a key area of research with profound implications for various human-centric applications. Context awareness refers to a system’s ability to utilize situational information, or context, to enhance and provide more relevant services [1]. Unlike human-to-human interaction, where implicit context can naturally enrich conversations, human–computer interaction often lacks this depth. By improving the detection and classification system’s access to context via RF sensing, we can enrich the user experience, especially in areas like handheld and ubiquitous computing, where user context is constantly changing. Context can be any information that defines the situation of a sensing target entity, such as a person or place. Context-aware computing uses this information to provide task-relevant services, automatically execute actions, or tag information for future use. Key challenges in this field include the creation of a unified taxonomy for context, developing supporting infrastructure, and identifying practical applications that enhance everyday interactions with computing systems.

With the advent of innovative RF devices operating at frequencies above 70 GHz and the proliferation of reconfigurable and software-defined radio devices, there exists a compelling opportunity to exploit RF sensing capabilities for context-aware applications. The availability of such devices enables fast experimentation at a low cost, facilitating the exploration of novel RF sensing techniques and algorithms. Moreover, recent advances in AI and DL have revolutionized the field of RF sensing by providing powerful tools for data analysis and decision-making. By leveraging AI and DL algorithms, researchers can extract valuable insights from RF signals in real-time, enabling robust object and motion detection and classification in dynamic environments. Importantly, the availability of powerful hardware, such as graphics processing units (GPUs) [2], has made it feasible to deploy complex AI algorithms on RF devices, thereby enabling real-time processing of RF data streams. This convergence of innovative RF devices, advanced AI techniques, and powerful hardware underscores the timeliness and importance of addressing context-aware RF-based sensing for detection and classification. The integration of RF sensing with AI-driven algorithms holds the potential to bring forth the next age of a wide range of applications, including autonomous vehicles, smart cities, healthcare systems, and environmental monitoring [3]. By harnessing the capabilities of RF sensing in conjunction with AI, researchers can develop context-aware systems that enhance situational awareness, improve decision-making, and ultimately enhance the quality of life for individuals in various human-centric environments.

Hence, the primary goal of this survey is to identify and evaluate AI and ML techniques that are particularly suited for handling RF signals in the context of object and motion detection and classification. By systematically reviewing the literature, we aim to identify the most promising AI/ML approaches that demonstrate efficacy in processing RF data streams and extracting meaningful information for context-aware applications. Additionally, this survey seeks to provide a comprehensive analysis of the pros and cons of existing techniques documented in the literature. By critically evaluating the strengths and limitations of different AI/ML methods, we aim to offer insights into their suitability for addressing the unique challenges associated with RF-based sensing. Furthermore, this survey aims to identify the existing challenges and gaps in the current state-of-the-art approaches and propose potential research directions to overcome these limitations. By highlighting areas for improvement and innovation, we aspire to guide future research efforts toward addressing key challenges and advancing the field of context-aware RF-based sensing.

At the heart of RF-based sensing applications lie object motion and posture detection, as well as classification [4]. Radar, being the most common implementation of RF-based sensing on a large scale, provides umpteen application examples in this regard, such as in domestic security, object detection, and classification frameworks, that serve to automatically identify possible concealed weapons without violating privacy, as seen in [5,6], as well as crowd threat detection, as alluded to in [7]. In aviation, RF sensing systems are employed to identify aircraft presence, malicious or otherwise, and ascertain their altitude. In autonomous vehicular control, RF sensing is employed for obstacle detection purposes, as explored in [8], to achieve safer road travel. In this use case, detection and classification could take the form of static and moving targets, such as in pedestrian detection frameworks [9] or parking assistance. In healthcare, they monitor vital signs, as depicted in [10] and changes in posture. The penultimate implementation is especially useful in palliative care, where the RF sensing-based system could be vital in the prediction and consequent mitigation of emergencies involving vulnerable subjects, as in [11]. Consequently, an additional objective of this survey is to provide researchers and practitioners with a thorough roadmap through the landscape of hitherto explored AI/ML techniques for context-aware RF-based sensing systems, enabling them to make informed decisions when designing and implementing these systems.

### 1.1. Related Surveys on RF Sensing and ML for Detection and Classification

In this subsection, we provide an overview of existing surveys and literature reviews on RF sensing, as well as ML for detection and classification, focusing on their contributions to the field and their relevance to our research. These related surveys offer valuable insights into the evolution, advancements, and challenges of RF sensing technologies, serving as foundational knowledge for our study. By synthesizing and analyzing the findings of these surveys, we aim to identify gaps, trends, and emerging research directions in the field of RF sensing. Additionally, we discuss the methodologies, scopes, and key findings of each survey, highlighting their contributions to the broader understanding of RF sensing techniques and applications. Through this comprehensive examination of related surveys, we establish a context for our research and provide readers with a comprehensive understanding of the current state of the art in RF sensing.

Existing surveys on context awareness, such as [12], have extensively covered this concept and its contribution to ubiquitous sensing. The study provides an extensive overview of context-aware computing within the IoT paradigm. It begins by outlining the rapid growth of sensor deployments worldwide and the resulting generation of vast amounts of sensor data. The paper emphasizes the importance of understanding this raw sensor data through context modeling, reasoning, and distribution. Context-aware computing techniques are explored as effective means of interpreting sensor data, with a focus on the context life cycle. The survey evaluates a subset of projects conducted over the last decade, providing insights into research and commercial solutions in context-aware computing for IoT applications. The research insights delved into comprised learning models in ML that are associated with context awareness. It concludes by highlighting lessons learned from past research, most notably on-demand modeling. The survey further mentions future research directions, including automated configuration, context discovery, context sharing, enhanced security and privacy, and sensing-as-a-service, addressing a wide range of techniques, methods, models, functionalities, systems, applications, and middleware solutions in the context-aware IoT domain.

The related surveys covered on RF-based sensing touch on the following application use cases:

1. Airborne RF Sensing: One of the most popular areas in which RF sensing has revolutionized operations in is airborne sensing. In this context, radar could be applied for threat detection, as seen in [13], which is a survey paper that offers a thorough analysis of RF sensing via radar and how it is used to identify, follow, and classify airborne threats. In this work, the vitality of radar technology is stressed in homeland security, aviation, and defense. The survey explains the basics of radar technology and how important it is for identifying and detecting aerial threats like drones, aircraft, and missiles, with the algorithms explored being divided into detection, tracking, and classification methodologies. The survey further examines different radar systems and elaborates on their benefits and drawbacks in various threat scenarios. These systems include mono-static, bi-static, and multistatic setups. The review explores the tracking algorithms and signal processing techniques used to preserve accurate target information over time. It also emphasizes classification methods like pattern recognition and waveform analysis to discern the nature of aerial threats. The survey paper emphasizes the importance of radar technology for national security and safety and concludes by proposing potential research directions for advancing radar-based threat detection and classification in a rapidly evolving technological landscape. Another key application of airborne RF sensing is overhead surveillance. For this purpose, the application of radar is not specifically for defense purposes and threat detection, as in [13], but can also be used in the large-scale tracking of deforestation activities, as in [14], or in tracking forest fires via overhead imagery, as depicted in studies such as [15]. To this effect, in [4], a comprehensive examination of DL-based object detection techniques and datasets specifically applied to the analysis of overhead imagery is provided. While exploring both active and passive radar sensing implementation, the paper highlights the importance of object detection in aerial and satellite imagery and discusses the unique challenges involved. The paper covers the basics of DL for object detection. A range of DL approaches, including faster region CNNs (Faster R-CNNs), you only look once (YOLO), single-shot detector (SSD), and others, are hence reviewed, analyzing their suitability and performance in the context of overhead imagery. Furthermore, the survey discusses various datasets commonly used for training and evaluating object detection models in this domain. It also addressed evaluation metrics specific to aerial and satellite imagery, offering insights into how performance is measured. Similarly, in [13], the paper identifies current challenges and proposes potential directions for future research in the field, emphasizing the continued importance of DL-based object detection for overhead imagery analysis. This survey is a prime example of a deep dive into the state of the art of radar applications in aviation and aerial detection, as well as object classification, while also providing insight on how the mathematical fundamentals are adapted to deal with the ever-increasing size of datasets.
2. RF Sensing for Automotive Use: On the other hand, survey papers, such as [16], further provides an in-depth exploration of the modeling aspects of automotive radar sensors, with a specific focus on their application in virtual testing and validation for automated driving systems. This particular survey comprehensively reviews a substantial number of papers, delving into the latest research in this domain. It highlights the significance of radar sensor modeling in the development of autonomous vehicles and the critical role it plays in virtual testing and validation. The paper also identifies several current research challenges, including the necessity for highly accurate radar sensor models capable of replicating complex real-world scenarios, the complexity of modeling interactions between multiple radar sensors, and the need to address issues related to interference, diffraction, and reflections within a virtual testing environment. Furthermore, the survey outlines various future research topics to address these challenges and advance the field. These include the refinement of radar sensor modeling for complex scenarios, enhancements in real-time simulation capabilities, and the integration of sensor fusion techniques to improve the accuracy and reliability of virtual tests for automated driving systems. The survey paper provides a comprehensive understanding of the state of the art in automotive radar sensor modeling and its application in automated driving systems’ validation.
3. Collaboration of RF Sensing and Other Communications: In existing surveys, the collaboration of RF sensing with other communication systems has been further touted in proposed solutions for many daily life issues. Most notably, much research has been done on the joint working of radar and mobile networks, for instance, in [17]. In this regard, mobile networks provide the ambient signals that passive radar systems rely on for operation [18]. Subsequently, as stated in [17], mobile networks, or other wireless communication infrastructures, require modifications in their protocols to accommodate these advancements. Further on this, in [19], the need for adjustments to be made to cater to the sharing of spectral resources, brought about by radar–mobile collaboration, is studied. Moreover, in [20], the study emphasizes on the need for current radar systems to dynamically modify transmission waveforms and operating frequency bands depending on real-time information to accommodate the increased functionality of radar as a direct result of radar–mobile collaboration.

The existing literature also comprises surveys on ML techniques for object detection and classification, aiming to provide a comprehensive overview of the state of the art in this rapidly evolving field of context-aware learning. These related surveys offer valuable insights into the various methodologies, algorithms, and applications of ML in object detection and classification tasks. By synthesizing the findings of these surveys, we identify common trends, challenges, and emerging research directions that inform our investigation. This analysis establishes a context for our research and highlights the contributions of existing studies to the advancement of ML-based approaches for object detection and classification.

Recent surveys on ML advancements for context-aware detection and classification, such as [21], provide a comprehensive overview of the integration of ML methods within context-aware middlewares (CAMs) for human activity recognition (HAR). HAR, a fundamental aspect of various domains such as ambient intelligence, smart cities, and e-health, involves inferring user activities from sensor data or context information. ML techniques enable more accurate activity recognition and anticipation compared to other paradigms. CAMs offer a flexible infrastructure for integrating diverse sensors and devices, facilitating the development of software solutions in sensor-rich environments. The survey systematically reviews the use of ML for HAR in CAMs, addressing key research questions related to context reasoners, ML algorithms, real-time data processing, and HAR scenarios. While ML presents promising approaches for HAR, including batch learning and data stream learning, challenges remain in addressing concept drift, adapting inference models, and integrating ML capabilities as services in CAMs. Future research directions include addressing these gaps to enhance the effectiveness and scalability of ML-based HAR solutions within CAMs. Other research works have further explored the contribution of DL to RF sensing frameworks. In this respect, ref. [22] explores the intersection of RF sensing and DL, highlighting its growing popularity due to its pervasiveness, low cost, and privacy preservation features. Despite its potential, RF sensing faces challenges such as multipath and interference. To address these challenges, the paper proposes leveraging DL to establish direct mappings from the RF domain to target domains, circumventing the need for complex RF physical modeling. The article introduces a four-layer framework for deep-interpreted RF sensing, comprising physical, backbone, generalization, and application layers. This layered approach provides a systematic methodology for designing deep RF sensing systems, enhancing generalizability and facilitating flexible applicability. The paper not only offers insights into the current state of RF sensing enhanced by DL but also points toward future research directions and promising advancements in the field.

### 1.2. Contributions

The main objective of this thorough literature review survey is to provide a comprehensive understanding of the state of the art for context-aware RF sensing for detection and classification. We aim to identify prevailing trends, challenges, and the direction of future research in this dynamic field by thoroughly analyzing carefully chosen journal and conference papers.

A significant challenge in the elected field, as the state of the art indicates, is the critical need to enhance detection and classification capabilities even further. However, all this indicates a much lower pace in that area compared to improving detection and classification technologies.

This survey makes an effort to review the developments in accuracy-centered RF sensing approaches, as well as emphasizes the need for the consideration of context in the design of such systems. Such paradigms, which allow for operability under distinct environmental, user, and cross-sectional conditions, are presented in a way that aids us in coming one step closer to the balance between accuracy and practicality. From this perspective, we have provided a possibility for augmenting existing methods in consideration of their scalability and context sensitivity, since now they are required to be applicable in dynamic and resource-limited situations. Such change is particularly important as the demand for RF sensing systems increases in healthcare and smart homes and other emerging fields such as autonomous systems’ operations.

The overarching contribution can be further decomposed into the proffered criteria followed in the systematic literature review of the state of the art in context-aware RF sensing, as well as ML for detection and classification. To gain a comprehensive understanding of the state-of-the-art techniques and advancements in the field of context-aware RF sensing, several key aspects need to be considered in the systematic literature review:

- Type of Physical Sensing (Active or Passive): Active sensing involves the emission of signals by the sensing device, such as frequency-modulated continuous wave (FMCW) radar, which actively sends out radio waves and measures the reflections from objects to detect and classify them. This approach is often more robust to environmental noise and can provide high-resolution data. Passive sensing utilizes existing ambient signals, such as WiFi channel state information (CSI), without actively emitting any signals. This method is less intrusive and can leverage existing infrastructure, but it may be more susceptible to interference and noise.
- Input Data Domain (Time or Frequency): Time domain data are analyzed based on the time variation of the signal. This approach can capture the temporal dynamics of the signal, making it suitable for applications where time-based patterns are crucial, such as motion detection. Frequency domain data are analyzed based on the frequency components of the signal, often using techniques like Fourier Transform. This method can be effective in identifying periodicities and distinguishing between different types of movements or objects based on their spectral characteristics.
- Spatial Diversity: It involves the use of multiple sensing points or antennas to gather spatially diverse data. This can enhance the sensing system’s ability to resolve objects in space and improve detection accuracy. Techniques like multiple input, multiple output (MIMO) leverage spatial diversity to enhance performance.
- Nature of Features Used (Raw or Statistical): Raw data implementations directly use the captured signals without significant preprocessing. This approach can retain all the information in the signal but may require more complex models to interpret the data accurately. Implementations using statistical features involve extracting meaningful features from the raw data, such as mean, variance, skewness, or higher-order statistics. This can simplify the data and highlight important characteristics, making it easier for machine learning models to perform classification.
- Classification Method (ML/DL Techniques Implemented): The choice of machine learning or deep learning algorithms for detection and classification is crucial, with common techniques including traditional ML in the form of kNN, SVM, and Random Forests and DL in the form of CNNs, RNNs, and Transformer models. These techniques can automatically learn complex feature representations from the data and improve classification accuracy.
- Accuracy/Evaluation Metrics Adopted: Common metrics include accuracy, precision, recall, F1 score, and area under the receiver operating characteristic curve (AUC-ROC). These metrics provide a quantitative measure of the model’s performance and help compare different approaches.

Examination of the state of the art regarding ML advancements for context-aware detection and classification encompasses data input stream types, ML algorithms, and DL models. Additionally, it scrutinizes the accuracy achieved by these methods and their adaptability across diverse contexts, coupled with proposed suggestions for how these methods can be improved to further streamline the systems. To this end, the following key aspects were considered in the systematic literature review:

- Objective and Scope: Clearly understand the objectives of the study, whether the focus is on a specific type of object or a broad range. Identify the scope of the research, such as indoor vs. outdoor detection, real-time applications, or specific domains like medical imaging, security/intruder detection, or autonomously driven vehicles.
- Sensor/Input Modality: Determine the sensor modality used for object detection. Common modalities include RF modes (of which radar is an implementation), lidar, acoustic sensors, vision-based (traditional RGB, infrared, stereo, time-of-flight, pan–tilt–zoom, and depth cameras), or a combination of multiple sensors.
- Dataset and Evaluation Metrics: Examine the datasets used for training and evaluation. A diverse and representative dataset is crucial for robust model performance, but the size of the dataset should not be overly prioritized, since computational time would be compromised. Additionally, understand the evaluation metrics employed, such as precision, recall, F1 score, or intersection over union (IoU), to gauge the model’s effectiveness.
- Deep Learning Architectures: Identify the DL architectures utilized, such as CNNs, region-based CNNs (R-CNNs), single-shot multibox detectors (SSDs), or YOLO. Understanding these architectures will provide insights into the model’s design.
- Training Strategies: Explore the training strategies employed, including transfer learning, fine-tuning, or training from scratch. Transfer learning from pretrained models on large datasets is common for object detection.
- Data Augmentation Techniques: Consider the data augmentation techniques applied during training to improve model generalization, especially in scenarios where labeled data are limited.
- Real-Time Performance: If applicable, assess the real-time performance of the proposed object detection system. Consider the processing speed and hardware requirements for deployment in real-world applications. This “feature” should be tested or benchmarked against the best- and worst-case scenarios.
- Challenges and Limitations: Understand the challenges addressed by the research and any limitations acknowledged by the authors. This provides a realistic perspective on the applicability of the proposed method.
- Comparisons with Existing Methods: Look for comparative analyses with existing object detection and classification methods. Benchmarking against established algorithms helps assess the novelty and efficacy of the proposed approach.
- Applications: Consider the applications and industries targeted by the research. Object detection and classification have diverse applications, including autonomous vehicles, surveillance, medical imaging, palliative care, robotics, and more.
- Recent Trends and Future Directions: Investigate whether the study discusses recent trends and emerging technologies or suggests future directions for research in object detection and classification.

### 1.3. Structure

The paper is thus split up into distinct sections, each of which focuses on a different facet of the aforementioned building blocks. Section 2 covers the fundamentals of context-aware RF-based sensing systems as seen in the current state of the art. The succeeding section, Section 3, covers the state of the art of context-aware RF sensing applications and use cases; this entails both existing use cases covered in scientific works and potential future use cases. Section 4 touches on ML advancements for context-aware sensing, delving into works implementing traditional learning, convolutional networks, DL, and advanced learning methodologies to supplement RF sensing frameworks. We then cover the challenges to RF sensing and accompanying proposed solutions present in the existing literature in Section 5 before suggesting some future research directions in Section 6 (this section is more inclined toward signal acquisition (low level) rather than use cases (high level)).

**Figure 1:**
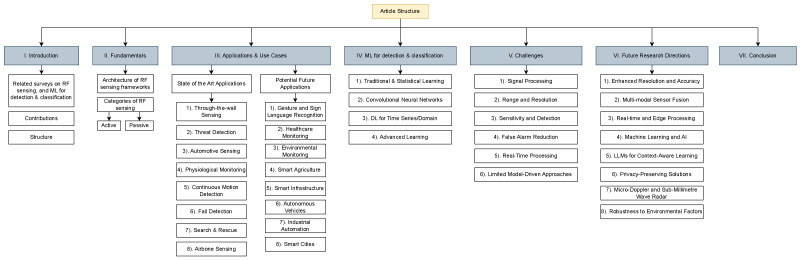
Illustrative diagram of the survey’s structure.

Figure 1 shows an elaborate chart depicting the structure of this survey.

## 2. Fundamentals

In this section, we delve into the foundational concepts underlying context awareness in the realm of RF sensing. Here, we explore the literature on the fundamental principles that govern RF sensing technologies and their integration with context-aware systems. This section provides an essential background for understanding the state of the art on how RF sensors capture environmental information, including radio signal propagation, modulation techniques, and signal processing methods. By elucidating these core principles, we aim to lay the groundwork for comprehending the intricate interplay between RF sensing and context awareness in the literature, paving the way for a deeper exploration of the subject in subsequent sections.

### 2.1. Architecture of RF Sensing Frameworks

**Figure 2:**
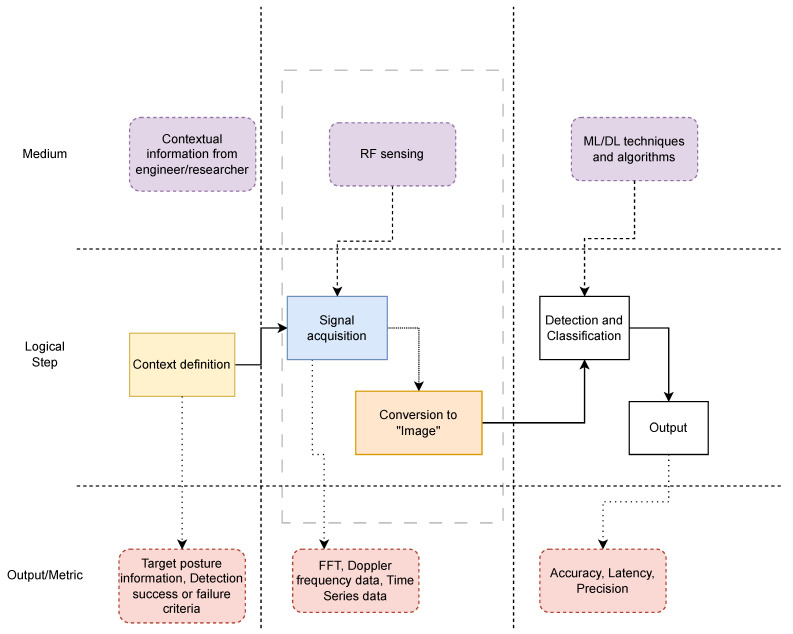
Logical flow of RF-sensing-based detection and classification framework (read top–down).

Figure 2 depicts the generic conceptual architecture of a context-aware RF-sensing-based detection and classification framework present across the existing literature.

This is the product of an amalgamation of the logical flow found in the studies considered in this survey. The endeavor is divided into three main logical steps depending on what medium is required to accomplish each step. These steps are as follows:

1. Context Definition: This is the most important step of the logical chain, as it is entirely dependent on the researchers’ understanding of the problem definition, i.e., the starting point of the study as regards what the sensing environment entails, what is defined as “interesting”, and what is not [23]. Taking the example of a fall detection framework, such as the one proposed in [24], this analysis governs what postures are defined as harmful and what postures are defined as harmless. In this case, context definition would also involve the target demographic of the proposed framework. Fall detection schema are likely to be geared toward detecting and classifying postures in elderly or invalid users. This means that the targets are likely to be moving at a much slower speed than normal, giving the people defining the context more leeway than if the schema were to be applied for crowd threat detection, as is the case in [7]. Context definition further entails outlining the success or failure criteria of the detection and classification framework, which is an endeavor that could have varying consequences in varying contexts. Take, for instance, the case studied in [25] where passive sensing was used for mine detection. In this event, the margin of error when defining what counts as a “positive” detection would be a lot slimmer than, say, in the case of [26], where RF sensing was applied for hand gesture recognition to enhance human–computer interaction for disabled users, since the former may involve catastrophic loss of life if detection is missed due to an inaccurate threshold definition. Furthermore, context definition determines the type of signal acquisition mechanism to be implemented, whether active or passive, and whether to use time or frequency domain data. For instance, in military operations, active sensing might be more accurate but might not offer as much discretion as passive sensing. On the other hand, passive sensing frameworks may be more susceptible to interference, since the signal source is not within one’s control. All this stems from a proper context definition.
2. Signal Acquisition: In this step, the logic can be further divided into two stages: the signal acquisition phase and the image conversion stage. In the signal acquisition phase, the primary objective is to capture relevant electromagnetic signals emitted (in the case of active sensing) or reflected by the target objects or subjects within the sensing environment (in the case of passive sensing). This process involves deploying RF sensors or transceivers strategically to cover the desired area effectively. The sensors receive the transmitted RF signals and convert them into electrical signals for further processing. Factors such as sensor placement, orientation, and antenna design play crucial roles in optimizing signal reception and quality. Additionally, considerations such as sampling rate, bandwidth, and signal-to-noise ratio are essential for ensuring accurate and reliable signal acquisition. The acquired signals are then preprocessed to remove noise, enhance signal quality, and extract relevant features for subsequent analysis and classification tasks. Overall, the signal acquisition phase lays the foundation for obtaining high-quality data inputs essential for the successful operation of RF sensing-based detection and classification systems. After signal acquisition, the signal frames are prepared for the detection and classification step. Since most of the ML techniques applied are geared toward image input, it is useful to convert the signal into a feature-based input. The resulting frequency domain data are often visualized as a spectrogram, which is a type of image that represents the intensity of different frequency components over time. By interpreting these spectrograms as images, powerful image processing techniques and DL models, such as CNNs, can be employed to analyze the patterns and features within the data. This approach utilizes the high resolution and rich information contained in the frequency spectrum to enhance the accuracy and robustness of object detection and classification in various RF sensing applications.
3. Detection and Classification: In the detection and classification phase of an RF sensing system, the primary objective is to identify and categorize the objects or phenomena of interest based on the acquired RF signals. This phase involves applying various signal processing and ML techniques to analyze the preprocessed data and extract relevant features indicative of different classes or categories (as covered extensively in Section 3.1.13). The choice of detection and classification methods depends on several factors, including the nature of the target objects or phenomena, the complexity of the sensing environment, the available computational resources, and the specific requirements of the use case. For example, simple thresholding or rule-based approaches may be sufficient for detecting and classifying binary states or predefined patterns, while more sophisticated ML algorithms such as DL networks may be necessary for handling complex non-linear learning models and dynamic scenarios with multiple classes and non-linear relationships (as covered extensively in Section 4). Ultimately, the goal is to develop robust and accurate detection and classification models tailored to the specific context of the RF sensing application, ensuring reliable performance in real-world scenarios.

### 2.2. Categories of RF Sensing

Categories of RF sensing can be broadly divided into two main types: active sensing and passive sensing.

#### 2.2.1. Active Sensing

Active RF sensing involves the transmission of RF signals by a dedicated transmitter and the consequent reception of the reflected signals [27]. This method enables controlled interrogation of the environment and target objects. Examples of active RF sensing technologies include radar, time-of-flight sensors, ultra-wideband (UWB) sensing, RF identification, short-range near-field communication, and mmWave sensing. Radar systems use radio waves to detect the range, velocity, and angle of objects, making them suitable for applications such as target tracking, object detection, and environmental monitoring. UWB sensing utilizes short-duration pulses of RF energy spread across a wide spectrum. By analyzing the reflections of these pulses, UWB sensors can accurately measure distances, detect objects, and penetrate obstacles. UWB sensing is suitable for applications such as indoor positioning, asset tracking, and vital signs monitoring [28].

#### 2.2.2. Passive Sensing

Passive RF sensing relies on the reception and analysis of existing electromagnetic signals in the environment [29]. This approach leverages ambient RF signals, such as WiFi in [30], Bluetooth in [31], and cellular or smart device signals in [32], to infer information about the surroundings and detect relevant phenomena. Passive sensing systems capture and analyze the variations in signal characteristics caused by the presence of objects or activities of interest. By monitoring changes in signal strength, frequency, phase, and other parameters, passive sensing systems can detect motion, presence, and behavior patterns. Passive RF sensing is advantageous for its low power consumption, reduced complexity, and compatibility with existing infrastructure. Applications of passive sensing include occupancy detection, human activity recognition, and context-aware computing in smart environments.

Both active and passive RF sensing techniques offer unique advantages and are suited to different applications and deployment scenarios. By combining these approaches and leveraging their complementary strengths, context-aware RF sensing systems can achieve enhanced performance and flexibility in detection and classification tasks across a wide range of domains.

## 3. Context-Aware RF Sensing Applications and Use Cases

As elaborated in the introduction, RF sensing has had a pervasive effect on the advancement of a variety of practical applications. In this section, we will explore the literature alluding to the application of active and passive RF-based sensing, as well as object, posture, and motion detection and classification in the aforementioned domains. This approach is adopted for the literature study to culminate in the identification of research gaps to be filled to tie in the building blocks together.

At the highest level of abstraction, a generic detection and classification system would comprise hardware and software subcomponents. This survey consequently follows the same blueprint. The hardware element of the system consists of RF sensing for signal acquisition, either non-radar or radar (active or passive, both of which can be further decomposed into domain-specific applications), while the software element consists of, ideally, preliminary signal processing stages and DL techniques for classification. In this survey, the aforementioned decomposition is implicit in that each study presented its sensing implementation, coupled with their subsequent detection and classification accuracy values. This allows us to further implicitly perform comparisons, albeit at a high level, between the domains in which active and passive RF-based sensing, as applications of RF sensing, are implemented. It does no harm to present research studies whose purpose it is to exclusively explore the comparison between active and passive radar.

**Figure 3:**
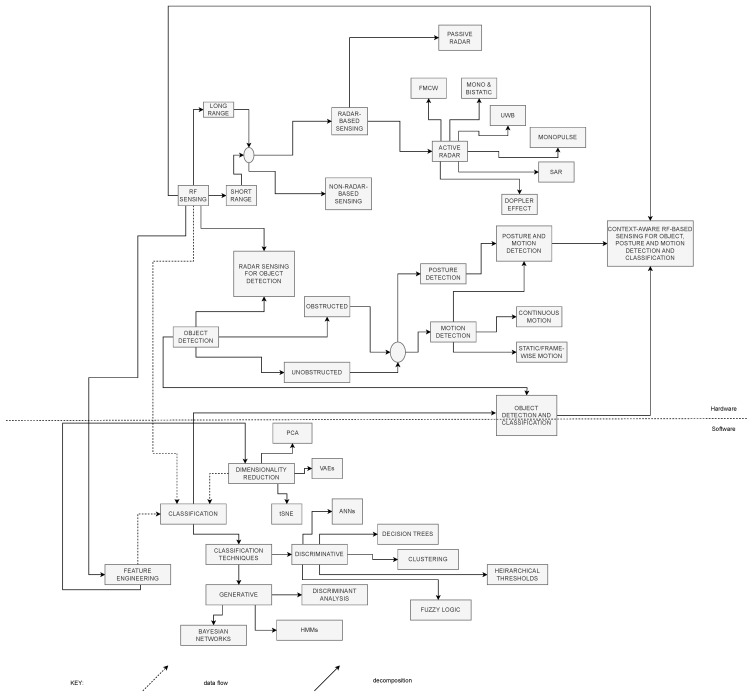
Taxonomy of topics considered in this literature review survey.

Figure 3 is a chart depicting the taxonomy of the topics covered in the papers considered in this literature review study to provide a foundation for “Context-Aware RF Sensing for Object, Motion, and Posture Detection and Classification”.

The taxonomy chart for the literature review survey offers a structured framework to navigate the complexities of this interdisciplinary field. Conceptually divided into two main sections, the chart delves into both the physical and software building blocks of the proposed solutions. At its root nodes, the taxonomy encompasses broad categories, namely, “RF Sensing Techniques” and “Classification”, reflecting the dual nature of the technologies involved. Within the RF Sensing Techniques category, the chart further decomposes into radar-based and non-radar-based sensing methods, reflecting the diversity of sensing modalities employed in the field. Radar-based methods, comprising both active and passive sensing approaches, include subtopics such as “Frequency-Modulated Continuous Wave (FMCW) Radar”, “Mono and Bi-Static Radar”, “Ultra-WideBand (UWB) Radar”, “Monopulse Radar”, “Doppler Radar”, and “Synthetic Aperture Radar (SAR) Imaging”, each with its own unique capabilities and domain of applications. Non-radar-based methods, on the other hand, encompass a wide range of approaches, including “Wi-Fi Sensing”, highlighting the versatility and adaptability of RF-based sensing technologies. Object detection, further divided into obstructed and unobstructed detection, emerges as one of the focal points of the survey, underscoring the importance of accurately identifying and classifying objects in various environmental conditions. Object detection can alternatively be decomposed into posture and motion detection, whereby the latter comprises continuous and static/frame-wise detection, the difference of which will be explored in latter analysis of the literature. Ultimately, the taxonomy converges at the ultimate topic of “Context-Aware RF-Based Sensing for Real-Time Object and Motion Detection and Classification”, capturing the overarching theme and objectives of the literature review. Through this comprehensive framework, the survey aims to provide a holistic understanding of the state of the art in RF sensing and AI/ML approaches, offering insights into the diverse methodologies, challenges, and opportunities in the field. Within the AI/ML Approaches category, nodes such as “Convolutional Neural Networks (CNNs)” and “Recurrent Neural Networks (RNNs)” represent specific ML methodologies employed for data analysis and classification. The decomposition of subtopics within each category leads to more specialized areas, including “Feature Extraction Techniques” and “Deep Learning Architectures”, reflecting the nuanced strategies employed to extract meaningful information from RF signals. Dashed arrows depict the flow of data between entities in the chart, illustrating the seamless integration of physical and software components in RF-based sensing systems. Regarding the software component, the dashed arrow indicating data flow to the “Classification” block depicts the contribution of feature engineering in extracting new features from existing data before modeling. The subsequent classification techniques are then divided into discriminative and generative classification methods. Classification additionally involves dimensionality reduction techniques, such as t-distributed stochastic neighborhood engineering (tSNE), principal component analysis (PCA), and variational auto-encoders (VAEs).

Following the taxonomy chart presented, the studies considered were each analyzed to deduce the following information: the physical sensing infrastructure used (whether active or passive sensing), the input data domain (whether the data collected were in the time or frequency domain, as well as information justifying this classification), how spatial diversity was achieved in the data collection phase of the study or would be achieved in the proposed sensing system (if it exists), the kind of features adopted (whether the data considered for detection/classification were raw or statistical), the ML (or otherwise) technology implemented for detection or classification, and the application domain of the study. Table 1 show a sample of the papers analyzed using these criteria.

### 3.1. State-of-the-Art Applications

#### 3.1.1. Sensing for Concealed Detection

One of the advantages that RF sensing possesses over other sensing mechanisms lies in its ability to be used in the non-intrusive detection of threats. In this context, the radar system implemented could take the form of a weapon detection scheme or a crowd detection scheme for spotting suspicious behavior by movement pattern analysis. Concerning concealed weapon detection, ref. [33] explored a method for detecting concealed weapons on individuals using W-band radar-based sensing technology that integrates both passive and active imaging. The proposed two-stage detection approach, which assesses the presence or absence of suspicious objects before identifying the object type, improves detection accuracy significantly. The methodology utilizes the temporal domain by capturing time-series images of a person’s movement. Active radar adds spatial diversity by using multiple radar imagery, whereas passive radar relies on ambient electromagnetic waves. The classification process examines time-series photos for potential threats by combining statistical and raw data. The experiment validates the approach’s effectiveness, emphasizing its accuracy when compared to methods solely focused on object type identification. The preceding study could be contrasted with [5], whereby the authors describe a concealed weapon detection system that relies heavily on active mmWave radar technology, which emits signals and analyzes their reflections. This system primarily makes use of raw data from radar signals, with the subsequent input data being in the time and frequency domains. Spatial diversity is achieved by combining active and passive sensors. The detection and classification of concealed weapons on individuals is based on analyzing radar reflections. While specific accuracy figures are not given, the paper emphasizes the effectiveness of combining active and passive sensors for concealed weapon detection, particularly in localized security applications, emphasizing the potential of such integrated systems in improving security and safety measures. On the detection of suspicious crowd activity, a method that employs RF-based machine vision to detect active shooters in crowded areas is presented in [7]. In this instance, we see a prime example of RF sensing implementation for ground security. Using RF technology, the physical sensing system collects data on the subjects’ surroundings and their respective movements while operating in the temporal domain. This is a callback to applications of context-aware motion prediction, as explored in [44]. Spatial diversity is then subsequently achieved via analysis of the subjects’ RF reflections. Utilizing raw data, this study focuses on RF signals and their properties. Machine vision techniques, i.e., Bayesian CNN, are then used in the classification process to identify anomalies in human movement patterns linked to active shooters. The study shows that RF-based machine vision has the potential to improve security in public areas by accurately identifying these dangers in challenging settings. Passive sensing could also come in handy when constructing below-ground (concealed) detection systems, e.g., for explosive mines, as in [25]. In such scenarios, active radar-based detectors, while accurate, run a very high risk of triggering the mines, potentially causing massive loss of lives. While comparatively less effective in locating mines, passive detectors do not pose the same risks. This study suggests a novel approach that takes into account soil type, magnetic anomaly, measurement height, and AI. This method shows promise for passive detectors, as it has been experimentally verified and achieved a success rate of 98.2% in mine detection implementing a heuristic kNN algorithm designed with fuzzy metrics. The model is additionally noteworthy for its ability to accurately classify five distinct mine types by using an ANN with an accuracy rate of 95.6%, thereby making a groundbreaking contribution to the field of mine classification. The success of the passive mine detector used in this paper shows the efficacy of the proposed approach while mitigating the risks associated with active detectors. Moreover, the work accomplished in [25] could be complemented by feature extraction to improve the accuracy of the detection system and to avoid mindless excavation [45]. Surveys, for instance [46], on ground-penetrating radar further support the foundations of the solution proposed above in [25], with the study examining the use of radar for mindful archaeological excavation. This widens the scope of influence of fields that are pervaded by RF sensing.

The RF sensing technologies that have heretofore been developed for concealed detection have shown great promise in terms of performance accuracy, given a variety of materials and barriers. Developers made the most notable contributions in this area of UWB radars, SAR, and passive RF systems, which allow resolution enhancement and light penetration ability without the line of sight. Such methods have been utilized in security, surveillance, and search-and-rescue missions, providing efficient yet non-invasive sensing capabilities. Despite the amount of work accomplished, substantial knowledge and implementation gaps still exist. Material complexity continues to pose significant challenges, as evidenced by numerous studies that underscore the limitations in accurately detecting or classifying objects concealed by metallic enclosures or multilayered barriers. Despite the advances in UWB and SAR systems, which have enhanced penetration, the dearth of robust modeling frameworks for material interactions engenders inconsistencies. Addressing these challenges necessitates the development of enhanced electromagnetic simulation models and adaptive algorithms that can account for variations in material properties in real time. Tradeoffs between resolution and range remain a necessity. High-resolution systems are limited in penetration depth, and long-range systems encounter difficulties with accuracy. The development of hybrid systems that dynamically adjust frequency bands or integrate multimodal sensing is a promising direction, as it allows for the optimization of performance for specific scenarios. Another recurring issue is the scarcity of large-scale, standardized datasets to benchmark concealed detection systems. Many studies rely on custom datasets that lack diversity in materials, object types, and environmental conditions. Collaborative efforts to create and share comprehensive datasets could significantly advance the field by enabling fair and reproducible comparisons across methodologies. The capacity to detect objects or activities through barriers additionally gives rise to privacy and ethical concerns, especially in civilian applications. To date, research has predominantly overlooked this dimension. Hence, future studies should incorporate privacy-preserving mechanisms, such as encrypting sensing data or constraining detection capabilities to authorized users, without compromising accuracy.

#### 3.1.2. Airborne Sensing

More interestingly, airborne radar can also be used for in-air threat detection, i.e., the detection of flying threats. In this respect, studies such as [47] address the growing challenge of detecting maneuverable flying targets, particularly in the context of active and passive radar. The rapid acceleration of these targets frequently causes blurring of the range-Doppler map, lowering the signal-to-noise ratio. The proposed non-parametric method estimated target acceleration on the range-Doppler map efficiently. The estimation algorithm presented in [47] outperformed existing methods in terms of speed, numerical stability, and simultaneous acceleration estimation for multiple targets by introducing a universal signal model applicable to both active frequency-modulated continuous wave radar and passive radar. The proposed technique is effective for autonomous real-time systems, contributing significantly to the accurate detection and parameter estimation of maneuvering aerial vehicles in both active and passive radar scenarios. This was subsequently validated by simulation tests and real-life radar signals observing a jet fighter and a drone. Airborne RF sensing can also encompass meteorological sensing for weather pattern analysis via cloud motion detection, as in [48], which introduced a deep learning-based cloud detection and classification algorithm designed for Himawari-8’s advanced Himawari imager (AHI) measurements from the geostationary satellite. This algorithm effectively combines passive RF sensing and consequently observed radiances with simulated clear-sky radiances to significantly improve cloud phase discrimination, particularly for optically thin clouds. Deep neural networks (DNNs) are employed to achieve simultaneous cloud detection, cloud phase classification, and multilayer cloud detection using multispectral observed radiances and simulated clear-sky radiances. Two DNN models were established for all-day and daytime-only applications, with reference labels derived from cloud profiling radar and cloud–aerosol lidar with orthogonal polarization merged cloud products. Validation using an independent dataset from 2017 demonstrated that both DNN models outperformed the official moderate resolution imaging spectroradiometer (MODIS) and AHI products in cloud detection and phase discrimination, particularly over land. For multilayer cloud detection, the probability of detection reached approximately 60% for the all-day model and increased to about 70% for the daytime model. This represents a substantial improvement compared to MODIS and AHI products. Additionally, the DNN models excelled in detecting optically thin cirrus clouds, which are often omitted by MODIS and AHI products. Furthermore, the DNN models exhibited superior capability in identifying mixed-phase clouds. These findings underscore the potential of DL-based algorithms for measurements from other similar instruments, providing a new level of accuracy and reliability in cloud detection and classification.

The state of the art in RF sensing for airborne applications, as demonstrated in the referenced literature, exhibits significant advancements in radar-based systems, signal processing techniques, and context-aware methodologies. These developments have led to enhanced detection, tracking, and classification of airborne targets, including drones, aircraft, and other aerial objects, across diverse environments. Noteworthy advancements in RF sensing technology for airborne applications include the integration of SAR for high-resolution imaging and phased-array systems for adaptive beamforming, which have been shown to enhance the precision and robustness of airborne RF sensing systems. Furthermore, ML models have been increasingly integrated to enhance feature extraction and object classification in complex scenarios.

However, several gaps remain evident, underscoring limitations in the current state of research and development. Most notably, the performance of airborne RF sensing is significantly affected by environmental factors such as atmospheric attenuation, precipitation, and electromagnetic noise from natural and man-made sources. While some studies address these issues through adaptive signal processing, the lack of universal solutions applicable across diverse weather conditions is a notable gap. To address these challenges, future research could explore hybrid approaches that integrate RF data with optical or infrared sensing, thereby enhancing resilience in adverse conditions. The selection of operating frequencies for airborne RF sensing also involves a tradeoff between resolution and range. For instance, higher frequencies (e.g., millimeter waves) offer enhanced resolution but are susceptible to atmospheric attenuation, while lower frequencies (e.g., L-band) are more robust but provide reduced spatial detail. The development of multiband RF sensing systems capable of dynamically adjusting operating frequencies based on environmental and operational context is a promising avenue for future research. Moreover, the integration of context-aware frameworks into airborne RF sensing is still in its infancy. While ML models have been deployed to enhance target classification, their adaptability to dynamically evolving airborne scenarios (e.g., congested airspace or rapidly shifting weather conditions) remains constrained. This underscores the necessity for research endeavors focused on the development of real-time learning systems capable of updating their models based on live data without necessitating extensive retraining. Many airborne sensing platforms, particularly drones, operate under stringent energy constraints. Current RF sensing technologies prioritize performance metrics such as accuracy and resolution, often overlooking energy efficiency. The advancement of low-power RF systems, equipped with efficient ML algorithms tailored to the unique requirements of airborne platforms, holds the potential to bridge this gap. On regulatory and ethical challenges, the proliferation of airborne RF sensing technologies introduces regulatory and ethical challenges, particularly concerning privacy and unauthorized surveillance. Existing studies have scarcely addressed these aspects in a comprehensive manner, thereby creating an opportunity to explore privacy-preserving mechanisms and protocols tailored for airborne RF sensing systems.

#### 3.1.3. Through-the-Wall RF Sensing

The previously mentioned non-intrusive nature of RF sensing additionally comes in handy for sensing operations that might require stealth, such as through-the-wall sensing for a variety of operations such as military and disaster recovery or search and rescue operations. With this in mind, ref. [34] introduced a novel approach for simultaneous through-the-wall 3D RF imaging and motion detection using radar. This system employs active radar technology, utilizing a synthetic aperture radar (SAR) with a stop-and-go trajectory. Being an active radar system, SAR operates by emitting radar signals and capturing their reflections. The input data are mainly in the time and frequency characteristics domains. Spatial diversity is then achieved through the stop-and-go trajectory within 30 s windows, enabling the collection of data from multiple angles. The method predominantly utilizes raw radar data for processing, focusing on the direct signals and their pertinent characteristics, with classification involving the analysis of Doppler frequency reflections to detect both the presence and movement of target subjects behind walls. The study reports promising accuracy in achieving through-wall 3D imaging and motion detection using as little as 3 s of acquired data, showcasing the potential of this technology for security and surveillance applications. Conversely, ref. [35] introduced a novel non-invasive through-the-wall human motion direction prediction system based on a single-input–single-output (SISO) communication channel model and an SNN. In this study, the use of radio signals and neural networks typically implies an active RF-based system. SISO modeling is implemented to classify unique human motion patterns embedded within received pilot radio signals. The research results demonstrate a prediction accuracy of approximately 89.13%. It contributes to the improvement of non-invasive human motion recognition systems, intrusion detection systems, and well-being and healthcare applications through enhanced processing techniques for monitoring and control. An additional contribution of this study is that it highlighted the use of feature extraction techniques, primarily PCA, in other studies exploring RF-based sensing for context-aware recognition in [49,50,51]. The latter study is further instrumental in that it implemented a CNN model with 8 complex human activities, namely, hug, pat, push, kick, high-five, handshake, bow, and boxing. This allows for context-aware transitional feature extraction as well, which has had tremendous contributions in video gaming, child abuse protection, elderly care, and smart surveillance fields. In contrast to the SISO-enabled model by [35], using UWB MIMO radar, an effective through-wall human pose reconstruction system was then presented in [52]. The technology creates 3D radar images of human targets through walls using active radar, due to its capability to both transmit and receive signals. Discrete 3D point data are then created from these radar images. With a low human pose reconstruction error of about 38.84 mm, a lightweight DL network converts the point data containing human body features into 3D pose coordinates. With $1.02\times 10^{6}$ parameters and $2.75\times 10^{9}$ floating point operations, the study focused on real-world applications that highlight UWB radar’s potential in situations such as surveillance or search and rescue. Another example of UWB radar implementation for through-the-wall sensing is presented in [36], which explored an open-source dataset that highlights the use of active RF sensing for non-contact human information detection and monitoring. This is especially useful for home palliative care, newborn care, and hospital physiological monitoring, among other applications. The dataset records human motion statuses, and a CNN-based approach for human motion status recognition is suggested in their work. The dataset is useful for human motion status recognition, as shown by the well-trained CNN’s recognition accuracy of more than 99.7% for three motion statuses. Even though the dataset was gathered in a straightforward setting, it nevertheless makes clear how important it is for UWB radar industry organizations to work together to produce more open-source datasets that will further the development of UWB through-wall radars. Through-the-wall sensing can also be achieved by passive sensing. For instance, in [53], a passive system for through-wall human sensing that utilizes Wi-Fi to achieve sensing was studied, with the raw input data being in the time and frequency domains, and several receivers were used to record reflections to achieve spatial diversity. Through-the-wall detection and classification were then made possible by analyzing the Doppler shifts brought on by human movement. Although precise accuracy numbers were not given, the paper emphasizes how well passive Wi-Fi radar detects movement and human presence behind walls. In applications like security and surveillance, this technology has great potential, especially in cases where non-invasive sensing is critical. Another common application scenario of motion sensing using passive radar is in a domestic setting for security or palliative care purposes. To this end, ref. [43] presented passive IoT radar (PIoTR), a system that achieves this goal by utilizing RF transmissions from IoT devices. With the ability to function with a variety of signal sources, including Wi-Fi and ISM bands, PIoTR computes phase shifts brought on by human motion and produces Doppler spectrograms as a result. Four commercial IoT devices for home use were used to evaluate the system, which provided both coarse- and fine-grained sensing modes based on signal strength. The findings highlight the potential of passive radar for occupancy and activity monitoring within IoT ecosystems, with PIoTR achieving an average of 91% accuracy in occupancy detection (coarse sensing) and 91.3% accuracy in activity recognition (fine-grained sensing).

#### 3.1.4. Crowd and Individual Threat Detection

In addition to the joint active and passive radar systems addressed in the preceding subsection, explicit active radar systems can also be implemented in security operations for all manner of threat detection endeavors. For instance, to address localized non-intrusive threat detection, an explicitly active radar-based system for probable active shooter detection was presented in [37]. The study introduced a method for remotely identifying potential active shooters with concealed rifles, focusing on radar micro-Doppler and range-Doppler signature analysis, with the input domain comprising time and frequency domain signals. By extracting distinctive features from the micro-Doppler and range-Doppler information of individuals engaged in suspicious activities, the study utilized an ANN for accurate activity classification. Results indicate an accuracy of 99.21% in distinguishing individuals carrying concealed rifles from other similar activities. Notably, the radar-based approach ensures privacy protection, as it does not involve sensitive visual images but still provides reliable detection capabilities via through-clothing detection. This emphasizes the efficacy of the proposed system while addressing privacy concerns. Alternatively, non-intrusive concealed weapon detection techniques could take the form of a dynamic antenna array, such as in [54], whereby the study presented a privacy-preserving active-radar-based weapon detection technique using an active 75 GHz rotational dynamic antenna array, ensuring personal privacy by sampling reduced frequency domain information. It employs an innovative method for concealed object detection, particularly handguns, by analyzing sharp spatial frequency features, implying that the data are collected in the frequency domain. The approach combines a dynamically rotated active interferometer (a device used to measure interference in waves to make precise measurements of various properties such as distance, wavelength, and frequency [55]) with arithmetic feature extraction and a threshold-based classifier to identify concealed handguns. Noise transmitters are used for active illumination, enabling frequency domain signal sampling, while the rotational dynamics sample a circle in the 2D frequency domain, capturing sharp-edge responses. Experimental results demonstrate effective concealed object detection, achieving a classification accuracy of 0.908 and an F1 score of 0.916 using four consecutive measurements. In a similar light, the study presented in [6] elaborated on RF sensing for the detection of hidden explosives, which is a crucial task for military scenarios whose streamlining could serve to save a lot of frontline personnel. Thanks to its penetrative nature and ability to pick up the motion of vibrating objects, radar technology offers a compelling way to spot concealed threats while maintaining privacy. The MiDSIX system is an active radar-based prototype designed to measure the micro-Doppler signal produced by loudspeakers that are driven by sound waves to detect hidden threats, as also exhibited in [6]. The paper highlights the MiDSIX system’s evaluation against replica improvised explosive devices hidden behind various common materials, even though it does not explicitly detail potential sources and effects of bias. This evaluation shows how well the system can identify hidden explosives and describe induced micro-Doppler signals. With the MiDSIX system, an adaptable and easily deployable method for concealed explosives detection enhances security measures. In contrast to [7], where active radar was used for suspicious human motion detection in crowded spaces, ref. [30] explored a passive bi-static radar system that uses Wi-Fi signals to find targets in areas with a lot of clutter by piggybacking on existing Wi-Fi signals. With the Wi-Fi signal data predominantly acquired in the time domain, the passive Wi-Fi sensing system has the advantages of reduced electromagnetic interference and covert operation. The paper presented a method based on a sparse range-Doppler representation of the received signal to address the problem of target detection, after which, a direct signal rejection algorithm mades use of this representation to separate the target from the clutter in the background. Furthermore, the proposed solution can detect targets walking in opposite directions.

Particularly in the areas of detecting concealed weapons, tracking crowd dynamics, and spotting suspicious activity, the state of the art in RF sensing for crowd and individual threat identification shows notable advancements. Methods like millimeter-wave (mmWave) imaging and UWB radar have demonstrated exceptional accuracy in identifying potential weapons and distinguishing individual behaviors in crowded areas. Real-time threat classification in RF systems has been improved using ML models, such as RNNs and CNNs. The complete implementation and dependability of these systems in practical situations are hampered by several gaps that still exist. Current systems frequently struggle with environmental interference, such as multipath effects and electromagnetic noise in urban areas. While mmWave devices enable high-resolution sensing, they are susceptible to environmental attenuation (e.g., by weather conditions or non-metallic materials), limiting their efficacy. To solve this, future research could investigate hybrid sensing systems that combine RF and non-RF modalities, such as optical or thermal imaging, to increase robustness. Furthermore, adaptive ML models capable of real-time recalibration to dynamic situations may improve system reliability. Many RF danger detection systems are designed for specific scenarios (e.g., airports or stadiums) and do not adapt to other contexts, such as outdoor public spaces or high-mobility areas. This lack of transferability can be attributed to an overreliance on scenario-specific datasets during model training. To solve this, creating vast, diverse, and standardized datasets covering multiple threat situations may increase model generalisability. Transfer learning techniques and domain adaptation procedures may also help systems adjust to novel contexts. High-resolution RF sensing frequently requires extensive signal processing and ML models, resulting in latency and computational overhead difficulties that impede real-time threat identification. Research into lightweight ML models, such as knowledge distillation and model quantization, may enable faster inference without sacrificing accuracy. Edge computing solutions may also distribute computational burden, assuring real-time responsiveness. Although many systems detect objects with great accuracy, they frequently do not use contextual information to determine whether the objects or behaviors they detect are real threats. For instance, depending on the situation, a concealed object may not always constitute a weapon. By including situational and environmental information in the detection pipeline, such as crowd density, movement patterns, and past behavior, context-aware RF sensing could be improved. Multimodal sensor fusion and graph-based models may help this endeavor even more.

#### 3.1.5. RF Sensing for Automotive Purposes

Another key area that has benefited immensely from the advancement of RF sensing is the automotive industry. Ranging from mapping and obstacle detection during autonomous and non-autonomous driving to assisted parking and fatigue detection, RF sensing has become ubiquitous in the average driving experience. In light of this, ref. [56] focused on using the probability hypothesis density (PHD) filter framework to create maps for autonomous vehicles and automotive functions using data collected via radar. With data collected in the time domain (we know this because time evolution was considered when implementing the Gaussian mixture PHD filter, implying that the data possibly consist of samples acquired at discrete time intervals) using active radar, the benefits of this framework include the avoidance of detection, data association, and track handling issues that arise with traditional multiple-target tracking techniques. Two innovations were made herein to address the algorithm’s complexity issues. First, the suggested an algorithm for data clustering to organize PHD components into clusters, improving the PHD filter’s measurement update. It also added a merging step to make the map representation simpler. Using multisensor radar data collected from public roads, the research shows that the resulting map can support applications such as autonomous driving by effectively describing the environment from a human-like perspective. This study demonstrates how useful coupling radar sensing and a PHD filter could be for mapping stationary objects. Regarding radar-assisted driving, an active vehicle safety system that combines several sensors for real-time obstacle detection and status classification, such as in [57] to improve road safety and collision warning, comes in handy. The system cited combines active radar technology with the ability to detect obstacles based on the time and frequency characteristics of radar signals. It also combines mmWave radar and stereo cameras to effectively detect possible threats in the driving environment. While mmWave radar compensates for camera limitations by focusing on distant and longitudinally moving objects, cameras excel at detecting nearby dynamic objects, offering rich information and sensitivity to lateral displacement. The paper describes a mmWave radar system for relative dynamic object detection and a camera detector that uses “error vectors” for dynamic class identification. An obstacle region of interest (ROI) map is created by integrating objects from both sensors using a UV disparity obstacle detection algorithm. Using the vehicle’s kinematic model as a basis, this map classifies objects into dynamic and relative categories by comparing them to a predetermined danger area. The technique was successful in improving traffic safety and lowering accident rates, even at high vehicle speeds. In comparison, ref. [58] presented a safety cruise control system for vehicular networks. This system ingeniously combines active radar sensing and vehicle-to-vehicle communication (V2V) to enhance active safety in traffic environments. It was more intelligent and context-aware than the one proposed in [57], since it utilizes radar recognition algorithms and V2V communication to detect surrounding vehicles and their behaviors. Moreover, the paper introduced a connected cruise control (CCC) framework that considered the merging behavior probability of surrounding vehicles. This framework utilizes a combination of fuzzy support vector machines (SVMs) and sliding window detection to determine the merging potential of adjacent vehicles. It also incorporates a cruise distance strategy, which considers merging behavior probabilities, and applies it to the CCC system for early braking decisions. The longitudinal control of the host vehicle is achieved through acceleration control, considering both time delays and feedback errors. Field tests and simulations demonstrated the effectiveness of this comprehensive control framework in enhancing active safety by accurately detecting and responding to the lane-changing intentions of neighboring vehicles. RF sensing for automotive purposes is also instrumental in rough operating conditions like fog, smog, and rain, where these environmental conditions cause interference.

The reliability and precision of RF sensing systems are significantly impacted by environmental interference, which includes adverse weather conditions, electromagnetic noise, and multipath effects. This impact is particularly pronounced in the context of automotive applications. For instance, precipitation, such as rain, fog, and snow, can degrade signal quality and reduce detection accuracy. Despite this, RF sensing still outperforms other non-vision-based sensing mechanisms [59]. Additionally, urban environments with high signal reflections can introduce noise and false positives. To address these challenges, advanced signal processing techniques, such as adaptive filtering in [60,61] and beamforming [62], have been employed to enhance signal clarity and reduce noise. Furthermore, the integration of multiple sensors, such as LiDAR or cameras, through a multisensor fusion approach, has been demonstrated to enhance system robustness in harsh environments by leveraging the complementary sensing capabilities of each modality. ML models trained on diverse datasets have also emerged as a recent advancement, with the capacity to identify and adapt to interference patterns in real time, ensuring consistent performance across varying environmental scenarios. These methods demonstrate how RF sensing can maintain high reliability in dynamic and challenging automotive environments, thereby supporting applications like autonomous driving and advanced driver assistance systems.

Further on environmental interference, the extent of these effects varies across different frequency bands, influencing their suitability for specific applications. High-frequency bands, such as mmWave, are more susceptible to attenuation caused by rain, fog, or snow, which can degrade signal strength and reduce detection accuracy. Conversely, lower frequency bands, like sub-6 GHz, experience less attenuation, making them more reliable in adverse weather conditions but at the cost of reduced spatial resolution. Urban and industrial environments often introduce electromagnetic interference from other devices, which can distort RF signals. Lower frequency bands are more vulnerable to such interference due to their longer wavelengths, whereas higher frequencies are less affected but require sophisticated filtering and shielding techniques. Multipath interference, on the other hand, is a challenge across all frequency bands. Higher frequencies are particularly affected due to their shorter wavelengths and higher susceptibility to signal reflections. Techniques like beamforming, channel estimation, and advanced machine learning algorithms have been employed to mitigate these effects. To mitigate this, selecting optimal operating frequencies depends on the application requirements. For example, autonomous vehicles often benefit from mmWave for high-resolution sensing in short ranges but may rely on sub-6 GHz for long-range robustness under poor weather conditions. Combining frequency bands or fusing data from multiple sensors is an emerging trend to ensure reliability in diverse environments.

Akin to active RF sensing, several instances exist of passive RF sensing implementation in driving systems to improve both operators’ and pedestrians’ safety. In this regard [63], offers a cutting-edge fix dubbed HDSpeed that employs smartphone passive acoustic sensors to measure vehicle speed. The system extracts different features from gasoline-powered vehicles (GVs) and electric vehicles (EVs) by examining the relationship between acoustic patterns and vehicle speed. For the EV and GV models, it uses CNNs and LSTM networks, respectively. HDSpeed uses active acoustic sensing to measure the change in distance between the smartphone and passing vehicles to obtain fine-grained speed data. By taking into account previously identified speed segments, the system’s detection technique lessens interference from surrounding objects. The accuracy of HDSpeed was demonstrated by extensive real-world experiments, which yielded an average error of 2.17 km/h, highlighting its potential to enhance road safety where traditional infrastructure is lacking. Pedestrian safety can also be accomplished via collision avoidance, as seen in [9], whereby a system for capacitive sensing intended for the same purpose of ensuring pedestrian safety was explored. The main component of this system is a passive capacitive sensor that is built into the front bumper of the car. The purpose of the sensor is to identify oncoming pedestrians. It accomplishes this by projecting a forward-extending electric field, into which a pedestrian incursion induces a capacitive coupling between the sensor and the object. Every millisecond, the sensor detects changes in capacitance and stores the last 100 acquisitions for later analysis. After that, a predetermined pedestrian signature created in the lab is compared to this history. The protection systems kick in when a pedestrian gets within thirty centimeters of the car and a collision is about to happen. This innovative capacitive sensor not only enhances pedestrian safety but also offers potential applications in low-speed parking assistance.

#### 3.1.6. RF Sensing for Physiological Monitoring

As an augmentation to the role of radar in the advancement of the vehicular industry, studies such as [64] address the simultaneous real-time detection of multiple physiological signals in drivers. Following this, body motion, breathing, and heartbeat, in the context of using UWB active radar for life detection and vital signal monitoring, have been considered for vehicle driver health monitoring. The peculiar characteristics of UWB radar echo present challenges, as body motion signals frequently predominate and cause interference. Because of their dynamic spatial positions, it may be difficult to implement traditional fixed feature detection techniques for the detection of movable physiological features. The paper introduced a novel multifeature alignment two-layer ensemble empirical mode decomposition method to address these problems and obtain accurate physiological data. Simulations and experiments show that this method reliably extracts breathing and heart rates from human subjects who are in a slightly inclined position—the average seated position of a driver—in both static and dynamic scenarios while also effectively reducing bias from body motion. This technique is a vital contribution to fatigue detection while driving, which may lead to car crashes, loss of life, or property damage. Still on the use of RF sensing for physiological monitoring, ref. [10] explored the use of Doppler radar for non-contact vital sign detection in the presence of random body movement (RBM), which typically reduces the accuracy of detection. An active, continuous-wave Doppler radar vital sign detection system running at 5.8 GHz was used in the study. It created a novel technique to measure respiration rate during one-dimensional body motion by analyzing the frequency spectrum of vital sign signals under body motion. To precisely calculate the respiration rate, the technique extracts the direction of body motion and the new position of respiration peaks in the frequency spectrum. The suggested method achieved RR measurements with only a 7.15% error rate under significant 1D body motion according to experimental results, indicating its robustness against RBM and potential for non-contact vital sign detection. Physiological monitoring could also take the form of contact sensors, whose operation can be augmented by the use of UWB sensing, an implementation of radar, and RF sensing. One such example is presented in [65], which explored a solution for indoor, real-time, and passive vital signs monitoring using UWB and depth sensors. The system achieves context awareness and privacy preservation by integrating data from these aforementioned sensors. It operates in the time series domain, leveraging raw sensor data for vital signs extraction and activity recognition in, for instance, the detection of chest wall displacement due to respiration and heartbeat. This justifies the classification in the time domain. The evaluation includes detection accuracy and real-time performance metrics. ML architectures were utilized for processing, and the system was extensively tested in engineering and medical lab environments. The demonstration of VitalHub highlights its robustness and passive monitoring capabilities, offering a practical solution for longitudinal in-home vital signs monitoring. The solution’s performance was compared with that of a food-and-drug-administration-approved medical device, indicating its potential for real-world applications and future directions toward more efficient and context-aware sensing systems.

Similar to active RF sensing, passive RF sensing, which relies on illuminators of opportunity to achieve sensing [18], plays a role in developing lower-cost signs-of-life detection frameworks. In [66], the study presented a real-time phase extraction technique utilizing passive Wi-Fi radar to identify the minute motions of the chest that correspond with breathing. Wi-Fi is one of the most pervasive wireless technologies in use today, making the proposed solution comparatively easier to implement in contrast to frameworks that depend on active radar. Compared to conventional range-Doppler processing, this method stresses phase variation because of the modest amplitude and slight Doppler shift of these motions. The technique is based on time domain cross-correlation and includes a Hampel filter to help with outlier identification and removal. The research highlights the shortcomings of the traditional cross-ambiguity function for signs-of-life detection and first introduced the basic passive Wi-Fi model. The phase extraction procedure was then described, and finally, experimental results and analysis were presented. The study shows that breathing can be successfully detected in stationary subjects through walls and within rooms using both data broadcasts and Wi-Fi beacons. This further highlights the importance of joint effort of radar and other wireless communications and the numerous possibilities this collaboration has opened up [67]. Further on the latter, more studies, for instance, ref. [68], examined performance tradeoffs and presented a unified multistatic passive radar and communication system that harmonizes radar and communication functions. The system consists of a communication receiver, a passive radar receiver, and several transmitters. This system, which is characterized by power allocation optimization, efficiently balances information signal and radar waveform transmissions to maximize the probability of detection (PD) while guaranteeing that communication requirements are met. Since achieving exact optimization is analytically difficult, PD bounds were used to approximate the solution. The PD-rate regions for a given false alarm requirement were delineated to evaluate the system’s performance tradeoff in this paper. The study’s methodology incorporated simulations to validate that the proposed power allocation method performs better than suboptimal alternatives. The simulation results support this assertion.

This section’s reference to the latest advancements in RF sensing for physiological monitoring highlights the rapid progress in non-invasive, continuous, and context-aware health monitoring. Applications in healthcare have shown significant promise for methods that utilize radiofrequency (RF) waves to detect vital signs such as respiration, body movement, and heart rate. However, a critical analysis of these studies reveals shortcomings and challenges that must be addressed to improve applicability and reliability. Existing algorithms excel at tracking a single subject, but they struggle to detect interactions and overlapping signals in multisubject scenarios. One significant limitation is the lack of robust context-aware frameworks for identifying and distinguishing individuals in group settings. Researchers might explore multitarget tracking approaches and advanced spatial diversity techniques. In multisubject environments, contextual cues such as activity recognition or spatial positioning can also enhance system performance. The systems under review frequently have significant computing requirements, which restrict their use in environments with limited resources, such as wearable or Internet of Things devices. There is still much to learn about energy efficiency, especially in passive RF sensing systems. The scalability of RF-based physiological monitoring systems may be made possible by advancements in hardware design, such as low-power processing units and energy-efficient antenna systems. In situations with limitations, optimization methods for lightweight machine learning models can also improve deployment viability. The incorporation of RF-based physiological monitoring into current healthcare infrastructures, such as electronic health records and telemedicine platforms, is rarely discussed, despite the technology’s promise of stand-alone uses. Future studies could investigate interoperability standards and data fusion frameworks to facilitate the smooth integration of RF sensing data into larger healthcare systems. This will improve RF sensing’s usefulness in practical healthcare settings.

#### 3.1.7. RF Sensing for Continuous Motion Recognition

Probably the most rewarding area of application for RF sensing from a human-centric perspective, sensing for motion detection has also become a staple in the healthcare and security sectors. In this regard, extensive research into the implementation of active radar has been done. For instance, in [38], the study presented a novel system for continuous human motion recognition using an active FMCW radar system, making it well suited for real-life environments. The method, based on dynamic range-Doppler trajectory (DRDT), is designed to recognize sequences of human activities rather than individual actions. This introduces the issue of context awareness, which is very elusive but strengthens detection frameworks, as seen in other studies such as [69] and elaborated in surveys like [70]. Range-Doppler frames were derived from backscattered radar signals, and 28 features were extracted using DRDT (specifically, dynamic Doppler frequency, dynamic range change, and dynamic energy change) to track real-time human motions in time, range, and Doppler domains. Each human motion was separated using a peak search method. The system then utilized these features from range-Doppler, radar cross-section, and dispersion, combining them in a multidomain fusion approach to feed an ML classifier. This resulted in accurate and robust recognition, achieving an average classification accuracy of 91.9% in continuous motion recognition, thus demonstrating its practical feasibility and superiority. To complement this and to overcome the shortcomings of conventional mono-static radar systems that are susceptible to changes in human motion aspect angles, ref. [39] presented a novel method for classifying human activity using multistatic micro-Doppler radar signals, i.e., active radar. The suggested technique improves classification robustness by utilizing the multistatic radar system to capture various aspect angles and points of view of human targets, as well as including a two-layer convolutional PCA (for feature extraction) step before implementing an SVM classifier and diffusion layer. To divide the multistatic micro-Doppler data into intervals, it first estimates the aspect angles of target motion to several radar nodes. Data belonging to the same interval are fused. To obtain the classification results, which were, on average, as high as 94.72%, an adaptive weighted decision-level fusion technique is then used. This method enhances classification performance and facilitates data fusion by taking time-varying aspect angles into account, benefiting applications in surveillance and security. Additionally, motion detection has practical human-centric applications, such as those presented in [40] that address the problem of motion recognition for smart homes and palliative care, as well as surveillance in search and rescue missions. It uses a UWB radar system for non-contact human motion recognition. In this context, we can explore the versatility of UWB radar for long-range sensing, which is in contrast to the case in [64,71], whereby UWB was implemented for close quarters sensing. For detection, the system transmits signals using active radar, with the input data being in the time and frequency domains. Prescreening both in situ and non-in situ motions was conducted through the introduction of a multilayer classification method. For classification, features based on PCA and physical empirical features were extracted. With accuracy rates of up to 94.4% for in situ motions and 95.3% for non-in situ motions, the study shows promise for use in senior care and smart home scenarios. In this same regard, in [72], a compact 24 GHz Doppler radar system was used to investigate human movement detection, with a focus on its potential applications in surveillance, security, and biometrics. It analyzes human motion signals by using active radar technology with an integrated low-noise microwave amplifier. A two-channel CNN was used in the study, which included 1000 recordings from eight different motion classes, to extract features from time domain signals and continuous wavelet transform (CWT) spectrograms. There are two types of convolutional layers in one channel: one with 1D layers and the other with 2D layers. They demonstrate the potential of DL for human motion recognition with radar signals by achieving an overall classification accuracy rate of 98.85% for the dual-channel CNN model, using both noisy and de-noised signals. Ref. [73] addressed omnidirectional motion classification utilizing micro-Doppler signatures in mono-static active radar systems, which capture radar reflections off moving targets. Typically, Doppler-based classifiers are influenced by the target’s movement direction, making them “angle-sensitive”. To overcome this challenge, the study introduced an angle-insensitive classifier using a novel CNN. It also offers a clear definition of “angle sensitivity” and conducts experiments on datasets based on simulations and measurements. The results indicate that the proposed algorithm outperformed existing feature-based and deep learning-based approaches, resolving the angle sensitivity issue in micro-Doppler-based classification. This research not only demonstrates the classifier’s effectiveness but also its potential for improving motion recognition in radar-based remote sensing applications. Radar can additionally be utilized in human–computer interaction tasks; for example, in [74], the study used a dual-channel Doppler radar sensor to implement a computer input device that can be used as a remote mouse. For non-contact motion detection, the system makes use of short-range continuous-wave Doppler radar sensors, which results in an affordable solution. The sensor uses motion imaging algorithms to capture the movements of the human hand and fingers in a two-dimensional plane. This is achieved using direct conversion architecture, symmetric subcarrier modulation, and bandpass sampling techniques. The approach’s effectiveness was demonstrated by experimental results, which allowed for the recognition of unique motion signatures in human–computer interaction. While precise classification accuracy numbers were not given, experimental measurements confirmed the efficacy of the suggested architecture and algorithms. The study also highlights the potential of Doppler radar sensors in real-world applications for gesture recognition using an active radar system. In addition, ref. [75] presented a novel radar-on-a-chip system that operates at 24 GHz to provide real-time indoor presence detection. This implementation can also be considered for motion detection. Its ability to detect minute movements, such as typing in an office setting, was demonstrated. This system uses active radar sensing to identify subjects in indoor scenarios. The study evaluated its performance in contrast to a traditional PIR sensor used in intelligent lighting. Their complementarity was demonstrated by the fact that the PIR sensor primarily detects larger tangential movements, while the radar-on-a-chip can detect minimal movements as small as 1 cm in the radial direction. The radar-on-a-chip provides time domain data while operating at mmWave frequencies. At less than 200 ms, both sensors respond quickly. The system’s high classification accuracy for real-time indoor presence detection was reported in the paper, highlighting the potential of radar-on-a-chip for indoor presence detection. Ref. [76] also presented a mmWave radar-based non-invasive HARsystem. The system, which is based on active radar, uses enhanced voxelization, denoising, dual-view ML, and data augmentation to refine point clouds produced by processing mmWave signals. In physical environments, this method efficiently captures spatial–temporal information. It achieves accurate HAR by utilizing a dual-view CNN and taking advantage of the symmetry properties of radar rotations. Excellent results were obtained from the evaluation on a dataset containing seven distinct activities that was acquired from a mmWave radar platform, with the system reaching 97.61% accuracy in fall detection and 98% accuracy in activity classification.

The latest developments in RF sensing for continuous motion detection show notable progress in the use of RF signals for both object and human motion detection and classification. For motion detection jobs, methods including Doppler radar, FMCW radar, and millimeter-wave sensing have demonstrated significant potential in obtaining high accuracy. But a more thorough critical assessment of the current methods identifies several problems and knowledge gaps, opening the door for new lines of inquiry. For the extraction of motion features, recent research depends on conventional signal processing methods like wavelet analysis and the Short-Time Fourier Transform (STFT). Despite their computational efficiency, these approaches frequently have trouble handling intricate motion patterns or overlapping signals in settings with multiple users. An option is machine learning-based signal processing, especially deep learning models such as RNNs and CNNs. However, the actual implementation of such models is limited by the high processing cost and the absence of standard datasets. Creating lightweight, domain-specific machine learning models specifically for RF sensing is one way to potentially close this gap. Continuous motion detection is severely hampered by environmental interference, including multipath effects and electromagnetic noise. The majority of current techniques lack dynamic adaptability despite context-aware approaches’ attempts to address these problems by integrating sensory environment information. For instance, a lot of systems are based on static models that do not consider environmental changes. To develop models that dynamically adapt to changes in their environment, future research could concentrate on including adaptive learning strategies, such as reinforcement learning. While recent developments have shown that continuous motion detection is feasible in controlled conditions, it is still difficult to scale these systems to multiuser, real-world scenarios. Especially when users are close together, current methods frequently fall short in tracking motions or differentiating between numerous people. This restriction might be addressed by utilizing hybrid RF sensing modalities, such as integrating RF sensing with vision-based systems or by incorporating sophisticated beamforming algorithms. Continuous motion detection methods are limited in their use in applications with limited labeled data or quickly shifting motion patterns because they frequently need large amounts of data for training. Although they have shown promise in tackling this problem, few-shot learning and transfer learning techniques are still not well studied in the field of RF sensing. A crucial topic for further research is creating data-efficient models that generalize across many contexts without requiring a lot of retraining. A lot of cutting-edge techniques put detection accuracy first, frequently at the price of real-time performance and energy efficiency. For applications with limited resources, such wearable technology and Internet of Things (IoT) devices, this tradeoff is especially challenging. This gap can be closed and wider adoption made possible by creating energy-conscious algorithms and hardware accelerators tailored for RF motion sensing.

#### 3.1.8. RF Sensing Fall Detection

Previously covered works on RF sensing have emphasized its use cases in posture and motion detection and classification for fall detection. For this reason, expansive research into the streamlining of such frameworks has been done. For instance, ref. [77] presented findings toward the development of a generalized system that uses micro-Doppler signatures and FMCW radar to detect falls and HAR. This study employed an active RF sensing system and focused on how radar datasets for HAR can be applied and carried across various contexts and subjects, with volunteers from four demographics. For activity classification, a variety of ML algorithms were used, such as SVM, k-nearest neighbor (kNN), and the DL classifier GoogleNet, with transfer learning using the AlexNet algorithm extracting features from spectrograms being used to train and validate the SVM and kNN classifiers. The subsequent results demonstrated consistent test accuracy, ranging from 68.5% to 81%, across the four different locations when using the GoogleNet algorithm. Another instance of a comparable implementation in elderly care through fall detection is depicted in [78]. Micro-Doppler features obtained using CNN models such as Alex-Net, VGG-16-Net, and VGG-19-Net were utilized in this case. When combined with canonical correlation analysis, a channel attention network efficiently combines the extracted features by prioritizing discriminative information and eliminating redundant data. An SVM classifier was used to classify these fused features. With a 99.77% test accuracy, the suggested method performed better than current techniques, and there was a 2% to 4% improvement over the most recent state-of-the-art techniques. This innovation has the potential to improve the safety and well-being of senior users by improving the timely detection of falls. In [79], range-Doppler radars were combined with DL to create a fall detection system. While taking a special interest in feature engineering, this system automatically captures complex radar return properties by processing radar data in the time–frequency and range domains, and it greatly outperformed other approaches, such as PCA and approaches that use predefined, physically interpreted features, as well as focused on minimizing false alarms. Compared to these latter methods, the DL approach achieved a classification accuracy rate of over 90% in fall detection, making it a strong solution for human fall detection, particularly in assisted living and healthcare settings, where safety is of the utmost importance. On the fall prevention analysis front, ref. [11] compared DL and gait parameter-based approaches for the use of micro-Doppler radar (MDR) for gait classification, with an emphasis on fall risk and age-related differences. Two classification tasks were taken into consideration: identifying middle-aged (50s) and elderly (70s) adults for age-related gait analysis and separating elderly non-fallers from multiple fallers for fall risk detection. Simulated MDR signal data obtained from a motion capture gait dataset were utilized. The results show that, in comparison to DL using spectrogram images of MDR signals, the classification accuracy was higher when using gait parameters extracted from MDR signals with a support vector machine. The gait parameter-based method outperformed the DL approach with accuracies of 73% and 76%, respectively, achieving 79% accuracy for faller/non-faller classification and 82% accuracy for 50 s/70 s classification. These findings highlight how well gait parameter-based methods work with MDR to identify age-related gait abnormalities and fall risk analysis.

#### 3.1.9. RF Sensing for Search and Rescue

Overhead RF sensing also comes in handy in human motion and posture detection for facilitating urban rescue missions. In the event of a natural disaster, some victims may be stuck underneath the rubble of fallen buildings and such. Consequently, the see-through sensing capabilities of RF sensing deem it a viable candidate for such an endeavor. In [80], the study forthwith described a mobile FMCW radar system built for urban rescue missions, with a focus on human target identification in distress situations. The system includes precise phase-based vital sign tracking based on radar return phase data and micro-Doppler signature patterns. It accomplished 2D mapping of the scene using SAR imaging, automatically tagging human targets based on behaviors such as running, walking, and crawling. Experiment validations included azimuth human target recognition, multiple human target recognition, and outdoor 2D mapping. The results reveal that the system can gather vital signs and offer exact position information for 2D mapping, highlighting its potential for search-and-rescue applications. Furthermore, the work describes a technique for identifying 2D targets in the time domain utilizing mobile FMCW radar equipment. Overhead RF sensing implementations for search-and-rescue can also take the form of more personalized frameworks that are keen on on-the-body features to accomplish their goal. An example of this is visible in [81], where the study alternatively presented a Doppler radar-based human detection technique primarily intended for use in security, surveillance, and search-and-rescue scenarios. The system collects data using active radar and focuses on examining the physical attributes of targets. The method takes a spectrogram and extracts multiple features, such as limb motion frequency, stride, Doppler signal bandwidth, and signal strength distribution, from it instead of working with raw data. Notably, the system uses the individual stride characteristics of human subjects for classification, which is made feasible by the various target species’ differing kinematic signatures and leg lengths. Using fourfold cross-validation, the system used an SVM classifier trained on these features to detect human subjects with an astounding 96% accuracy.

#### 3.1.10. RF Sensing for Maritime Purposes

SAR is another noteworthy implementation of active radar, which is primarily used to achieve land, water, and airborne 3D surveillance, as explored in [82] with MIMO. An alternative implementation was exhibited in [83], where, with an emphasis on small-ship detection, the study discussed ship detection in polarimetric synthetic aperture radar (PolSAR) images. Radar signals in this case were sent out, and the subsequent reflected signals were recorded by the active PolSAR system. A novel method of ship detection was introduced by the proposed two-stage model. Using a feature known as SVVSO, which is based on intensity information with orientation angle compensation, sea clutter suppression is accomplished in the first stage. Using features like polarimetric intensity difference (PID) and non-surface degree (NsD), which are built from SVVSO and theoretical derivations, an enhancement process is used in the second stage to highlight ships. The two-stage-based FPAN method was introduced for ship detection by combining PID and NsD. Using four L-Band PolSAR datasets, the experimental results show that FPAN was more effective than other methods in detecting small ships, as measured by the figure-of-merit (FoM) and target-to-clutter ratio (TCR). Compared to the double spectrograph detection algorithm, FPAN improved the FoM and TCR by an average of 9.40% and 25.18%, respectively, with a time consumption of only 58.67% of the latter. In [84], the study assessed data enhancement techniques for active RF sensing systems, such as SAR, to detect ships. Manual interpretation of SAR data, which is usually in the time and frequency domains, can be difficult. The challenge of obtaining and deciphering SAR images has led researchers to explore neural networks as a means of improving ship detection in SAR surveys. The problem of inadequate training samples was addressed by using data enhancement techniques like rotation, shift, mirroring, and brightening to increase the size of the dataset. The study enhanced ship detection performance by combining multiple data enhancement techniques. The efficiency of these techniques in improving the accuracy of SAR-based ship detection was demonstrated by experiments carried out with the SSDD dataset. Large-scale RF sensing implementation could also be employed in detection schemes such as those presented in [14], which focused on the use of L-band SAR, a variation of SAR, for the early-stage detection of deforestation in tropical regions. Radar images of the Earth’s surface were obtained by L-band SAR, which functions in the microwave frequency range. The research used time series L-band SAR data to track and identify tropical deforestation activities. It processed raw SAR data and extracted pertinent features for early detection of deforestation activities in tropical regions, which is critical for environmental conservation and sustainability initiatives. Passive sensing has also had a major hand in maritime surveillance, with cases such as the development of magnetic anomaly detection (MAD) systems, as depicted in [85]. By monitoring minute variations in the Earth’s magnetic field brought on by ferrous materials, this system finds and tracks buried objects, shipwrecks, underwater vehicles, underwater explosives, and underwater submarines. While passive and active acoustic sensors are the main tools used in long-range surveillance for target detection and classification, MAD provides accurate final localization. For maritime surveillance, MAD is essential for increasing tracking precision. Through an understanding of the correlation between the kinematic parameters and the magnetic signature, a Bayesian framework is utilized to tackle the tracking issue. The paper integrated an airborne total-field sensor and uses a variety of non-linear filters for real-time single-surface target tracking. It also proved the efficiency of the filters in estimating kinematic states, permanent moments, and induced moments, and it set the posterior Cramér–Rao lower bound for MAD. The study emphasizes how important MAD is to improving airborne surveillance capabilities.

#### 3.1.11. RF Sensing for Environmental Conservation

Still on the contribution of radar to environmental safety and SAR implementation, ref. [86] explored the issue of marine oil spill identification, which poses significant environmental threats. Via fully PolSAR, this active radar system operates in the time domain, making it ideal for detecting oil spillage events. The proposed method enhances the accuracy and efficiency of oil spill identification using an active contour model (ACM). The key innovations include an optimal initial box boundary for the ACM and a noise suppression process. The ACM boundary selection is based on the median-level contour resembling an imaginary mountain. Additionally, noise reduction is achieved through a Gaussian smooth operator, ensuring the ACM’s stable evolution. Experimental results confirm the method’s effectiveness, with an overall identification accuracy of 99.8%. It outperformed traditional approaches and highlights the value of PolSAR in environmental monitoring.

#### 3.1.12. Gesture Recognition

In a different dimension of human–computer interaction, radar is also useful for a variety of gesture recognition operations, with notable works such as [41], where hand gesture recognition using active mode mmWave radar based on radar parameter spectra was investigated. The study highlights the possibility of using frequency data from radar measurements as features for gesture recognition by fusing radar theory with classification techniques. By creating six unique dynamic gestures, the research does away with the need for intricate definitions of start and endpoints. To increase precision and decrease accidental gesture interference, several motion range and detection coherence-related features were extracted. To effectively classify various gestures, the study implemented a decision tree classifier that was created based on experimental observations with an mmWave radar. This culminated in a classification accuracy of 90%. In comparison, ref. [42] further presented an active radar-based system for detecting mid-air gestures for digit writing using radio sensors, specifically a continuous-wave (CW) radar system. The system captures gestures in the time domain, focusing on human hand movements. It employs a CNN to process the radar data and classify the gestures. The CNN is trained to recognize the unique patterns created by the gestures as they interact with the radar waves. The system demonstrated promising accuracy, with a recognition rate of approximately 92%, showcasing its potential for use in applications in human–computer interaction and sign language recognition. This technology offers a non-contact and robust approach for capturing hand movements.

The cited cutting-edge research on RF sensing for gesture recognition has significantly advanced the use of radio frequency signals for accurate and non-intrusive computer system interaction, especially for people with accessibility requirements. Methods including FMCW radars, time-of-flight measurements, and Doppler shift analysis have demonstrated encouraging outcomes in capturing both large-scale body motions and fine-grained hand movements. Additionally, the incorporation of ML models, such as RNNs and CNNs, has improved contextual comprehension and the accuracy of gesture recognition. Nevertheless, several significant knowledge gaps and obstacles still exist, impeding the wider use of RF sensing for accessible computing. One major deficiency is the lack of extensive, varied, and openly accessible datasets dedicated to gesture recognition. The generalizability of trained models is limited by the lack of variation in user demographics, environmental conditions, and gesture kinds found in existing datasets. Future studies should concentrate on building extensive datasets that encompass a variety of users and scenarios to overcome this and enable more reliable and transportable models. Multipath effects, congested environments, and changing illumination are only a few examples of the environmental interference that many gesture recognition systems encounter. While some studies suggest multimodal sensing systems or sophisticated signal processing techniques, more research is required to create adaptive algorithms that can dynamically account for environmental variability without incurring a large computational cost. It is still difficult to achieve great accuracy in real-time performance, particularly for devices with limited resources. The usability of gesture recognition systems for accessible computing applications in the real world is frequently restricted by this tradeoff. Future research should investigate dimensionality reduction strategies, hardware accelerations (such as edge AI), and lightweight machine learning architectures that reduce latency while maintaining crucial classification features.

#### 3.1.13. Feature-Based Recognition

Feature-based recognition and feature engineering could significantly reduce the dimension of consideration of the detection and classification framework, translating to improvement in performance. Due to this, feature recognition is also a vital building block of data preprocessing. Feature-based recognition has been advanced by the implementation of feature engineering and extraction tools, such as VAEs, as in [87]. In this study, the objective of the experiment considered was to enhance real-time indoor detection of human postures using radar sensors, focusing on minimizing data while maintaining classification accuracy. The study leveraged time domain radar data as it captured variations in posture and activity over time and adopted raw data for feature extraction. VAEs were employed for feature engineering, with t-SNE and PCA used for dimensionality reduction. Five solutions were proposed and compared based on classification accuracy and computation time, showing classification accuracy exceeding 90%. Furthermore, the research reveals a tradeoff between dimensionality reduction and system performance. The findings also highlight the potential of radar-based systems combined with AI techniques for effective human posture detection in real-time applications, emphasizing the importance of balancing data preservation with computational efficiency for future improvements. In more specific practical scenarios, ref. [26] presented a feature-based hand gesture recognition system utilizing FMCW radar. The radar is equipped with spatial diversity to capture hand gestures with improved precision, and the input data are collected in the time domain. The system employs a temporal feature analysis technique to extract relevant information from radar signals over time. These temporal features are subsequently used for hand gesture classification through feature selection via a quantum-inspired evolutionary algorithm (QEA). The results from the proposed approach were then contrasted with those from classification using kNN and random forests algorithms, with the QEA-based algorithms outperforming the two, albeit slightly. The study highlights the potential of FMCW radar for contactless hand gesture recognition, which can be applied in human–computer interaction and healthcare. With the implementation of a 77 GHz FMCW MIMO radar, ref. [88] further presented a real-time multigesture recognition system. This active radar system utilizes MIMO capabilities to provide spatial diversity while gathering raw data in the time and frequency domains. For real-time multigesture classification, the system uses the dynamic DTW algorithm and feature-based gesture recognition. Novel applications of complementary metal oxide semiconductor radar technology have been made possible by technological advancements in this field. By overcoming the limitations of single-chip radar systems that are less expensive, the suggested solution achieved an astounding 96% accuracy for six gestures across eight users. With only 8.4% DSP cycles and 256 KiB of memory used on Texas Instruments’ AWR1642, the solution exhibits good performance while being resource-efficient. An additional instance of feature-based RF sensing implementation is apparent in [89], where the study presented a new method for generic target classification using mmWave FMCW radar systems. This active radar system uses range-fast Fourier transform (FFT) plots to extract raw data features and operates in the mmWave frequency range (77–81 GHz). Peak characteristics, width, area, standard deviation, and range of FFT plot peaks are some of these features. The use of ML models for classification, such as logistic regression and naive Bayes, was examined in this paper. The results point to logistic regression spotting a 86.9% classification accuracy. By going beyond range detection, this method increases the capabilities of mmWave radars and creates opportunities for use in autonomous traffic control systems, computer vision, and object recognition—particularly in ground station traffic management.

Alternatively, RF sensing could be amalgamated with other sensing frameworks, such as wireless sensory networks (WSNs), as accomplished in [90] for intruder detection. To improve their capabilities, these networks integrate traditional WSNs with active radar sensors. Radar sensors that effectively combine sensing and communication functionalities were designed using the integrated sensing and communication approach. Techniques for effectively disseminating data were pursued, particularly in regions without base station coverage. Storage nodes stored data from radar sensors, and mobile data collectors periodically collected these data to optimize network usage in the time domain. The network performance was analyzed using epidemiological modeling, and the radar sensor density was maximized for effective data dissemination. The analysis and optimization techniques were validated by the simulation results.

To this point, feature-based detection in the realm of RF sensing has shown notable progress. In order to improve recognition accuracy and flexibility to a variety of scenarios, numerous studies have investigated the merging of ML and DL techniques to automate feature extraction and categorisation. But even with these successes, there are still a number of important questions and problems that need to be looked into further. Although many research concentrate on increasing classification accuracy, they frequently make the assumption that contexts are static or well-defined, which restricts the adaptability of RF sensing systems in dynamic, real-world settings. Context-aware feature identification is still not well studied, particularly in crowded or multiuser environments. One of the biggest challenges is the ability to distinguish in real-time between users, activities, and environmental interferences. Although deep learning models frequently produce better results, it can be challenging to comprehend how particular variables affect categorization decisions due to their reliance on black-box designs. The use of RF sensing devices in vital applications where transparency is essential, like healthcare or security, is hampered by this lack of interpretability. The task-specific feature engineering that is often the emphasis of current techniques limits the transferability of learnt features across various applications or domains. Features designed for posture detection, for example, could not transfer well to other tasks like object classification or fall detection. Environmental elements like multipath effects, electromagnetic interference, and meteorological variables can still affect RF sensing devices. Although there are mitigating techniques, more research is needed to determine how they affect feature robustness in different contexts. The computational cost of feature extraction and classification methods continues to limit the real-time performance of feature-based recognition. Although they have been investigated, methods such as dimensionality reduction (e.g., PCA or t-SNE) frequently require balancing accuracy and latency.

The study of active RF sensing in present literature emphasizes its importance as a versatile and powerful technology. Active radar systems continue to prove their worth in a variety of applications, ranging from military to civil. Radar technology has evolved from low-level domains such as waveform design and signal processing to high-level adaptive features that contribute to increased target identification, classification, and tracking capabilities. The input data are mostly in the time and frequency domain, with validation being done primarily by simulation. Moreover, as the accuracy values show, the proposed radar-based frameworks outdo the existing systems, which emphasize the advancement of active RF sensing, establishing it as a must-have technology for applications ranging from surveillance to environmental monitoring.

### 3.2. Active Sensing vs. Passive Sensing

To this point, we have explored a myriad of application areas of RF sensing technology, either as part of or as an entire object and motion detection system. In the spirit of championing sustainability, it would be negligent not to explore the difference in application domains and/or situations between active and passive radar.

To this effect, this section presents the literature that studies a comprehensive examination of the differences in performance between active and passive radar systems. Active radar systems emit radio waves and receive their reflections to detect and locate objects, whereas passive radar systems use existing sources of RF radiation, such as TV or radio signals, to detect and track objects.

In [91], the study put forward the claim that because active radar systems have performance metrics that are well established and allow for control over transmitted signals, they have historically been used more extensively. They can function in a variety of settings and weather circumstances and offer excellent target detection accuracy and resolution. They can be identified, and their signals are jammed, though they are noticeable. On the other hand, passive RF systems, which make use of the ubiquitous electromagnetic signals in their surroundings, have become a viable substitute. This technology reduces electromagnetic interference and provides affordable solutions. They offer built-in resistance to jamming and are less noticeable. The authors stress that it is difficult to compare the performance of different radar systems because it depends on several variables, including the particular application, the sources of the signals, and the capabilities of the receiver. In general, active radars perform better than passive radars in terms of target discrimination, accuracy, and range.

Passive radars do, however, perform best in covert operations where it is essential to remain undetected, as exhibited in multiple examples in the literature [53]. One of the main arguments in [91] is that the proliferation of broadcast sources and advances in digital signal processing can greatly benefit passive radar systems, increasing their effectiveness and practicality in a variety of applications. This additionally means that the implementation costs for passive radar would be comparatively favorable. However, the implication of such a framework leaves one vulnerable to external “movable” parts, eloquently phrased as the “Multiple Points of Failure” problem. In this respect, active radar systems, in comparison, would be a lot more compact; hence, it would be easier to plan for failure. Still, passive radars are advantageous in tracking stealth targets that take advantage of low-observability techniques, border surveillance, and civilian air traffic monitoring. The conclusion in [53] emphasizes the need for context-specific comparisons between active and passive radar systems. Although passive sensing systems have certain advantages over active sensing systems, including the ability to operate covertly, lower cost, and resilience to electromagnetic environments, they might not perform as well in some situations. Overall, the paper provides valuable insights into the performance differences between active and passive RF systems, shedding light on their respective advantages and challenges in various scenarios.

Table 2 shows a comparison of active and passive radar in existing literature based on a few selected features.

The energy efficiency and power consumption of context-aware RF sensing systems vary significantly between active and passive sensing implementations, particularly in resource-constrained environments like IoT devices. Active RF sensing, such as mono-static or bi-static radar, requires dedicated transmitters to emit signals and receivers to process reflected signals. This dual-component operation often results in higher power consumption, as energy is needed to generate the RF signals and continuously process the reflected data. As a result, active sensing implementations can be less energy-efficient, making them less suitable for IoT devices that rely on battery power or limited energy sources.

In contrast, passive RF sensing leverages existing RF signals in the environment (e.g., Wi-Fi, Bluetooth, or cellular signals) for sensing tasks, eliminating the need for dedicated transmitters. This significantly reduces power consumption, as the system primarily focuses on receiving and analyzing ambient signals. Passive implementations are inherently more energy-efficient, making them ideal for IoT devices and other low-power applications. However, the tradeoff lies in their reliance on the availability and quality of external RF signals, which can impact sensing performance in some scenarios.

For both approaches, energy efficiency can be further enhanced by leveraging advanced signal processing techniques and lightweight machine learning models that reduce computational overhead. Edge computing and hardware accelerators like GPUs or TPUs can also help manage real-time processing demands without excessively draining power.

In summary, while active sensing provides robust performance in controlled settings, passive sensing offers superior energy efficiency for resource-constrained environments like IoT. This tradeoff highlights the importance of tailoring RF sensing implementations to specific deployment scenarios.

### 3.3. Potential Use Cases

RF sensing has the potential to revolutionize various fields and enable new applications in the future. Some potential future use cases of RF sensing include the following:

1. Healthcare Monitoring: RF sensors can be used for non-invasive health monitoring, including vital sign monitoring, sleep tracking, and fall detection [101]. These sensors can provide continuous, real-time monitoring of a patient’s health status without the need for physical contact. Additionally, improvements regarding IoT-AI integration could further streamline the functioning of these frameworks.
2. Environmental Monitoring: RF sensing can be employed for environmental monitoring applications, such as air quality monitoring, water quality monitoring, and pollution detection. RF sensors can detect and analyze environmental parameters remotely, enabling early detection of environmental hazards. RF sensing in this respect could also contribute to industries, e.g., fishing [102], thus increasing food security.
3. Smart Agriculture: RF sensing can contribute to precision agriculture by monitoring soil moisture levels, crop growth, and environmental conditions in real time [103,104]. These data can be used to optimize irrigation, fertilization, and pest control practices, leading to improved crop yields and resource efficiency. RF sensing advancements can also be geared toward providing low-cost solutions to bridge the gap between commercial and subsistence farming and avail higher-quality products for smaller-scale producers.
4. Smart Infrastructure: RF sensors can be deployed in infrastructure systems such as bridges, roads, and buildings to monitor structural health and detect anomalies in real time. This proactive approach can help prevent infrastructure failures and enhance public safety. Integration of RF sensors with IoT platforms allows for seamless data collection, processing, and visualization, enabling centralized monitoring and management of smart infrastructure networks [105].
5. Autonomous Vehicles: RF sensing technology is already used in autonomous vehicles for object detection and localization. In the future, advancements in RF sensing could further improve the accuracy and reliability of autonomous driving systems, enabling safer and more efficient transportation. RF sensors can facilitate communication between vehicles and infrastructure, as well as other road users, enabling cooperative driving strategies, traffic management optimization, and enhanced situational awareness for autonomous vehicles [106,107].
6. Industrial Automation: RF sensing can play a crucial role in industrial automation by monitoring equipment health, detecting defects, and optimizing manufacturing processes. RF sensors can provide valuable insights into machine performance and help minimize downtime and maintenance costs. RF sensors can be used to track the movement of raw materials, work in progress, and finished goods throughout the supply chain. This visibility enables better coordination between suppliers, manufacturers, and distributors, leading to reduced lead times and improved resource allocation [108].
7. Smart Cities: RF sensing can contribute to the development of smart cities by enabling various applications, including traffic management [109], waste management [110,111], and public safety. RF sensors can collect data from urban environments to improve resource allocation, enhance public services, and ensure sustainability.

## 4. ML for Context-Aware Detection and Classification

In this section, the literature that explores advancements in algorithms for ML aimed at object and posture detection and subsequent classification (irrespective of the implemented sensing modality) is covered. With this in mind, it would be vital to explore the different data input sensors and data domains, as well as the application area considered. The latter is especially important, seeing as the practical environment would determine what the preprocessing stage would comprise and the consequent effectiveness of the model upon detection and/or classification. Consider, for instance, the case of a posture detection and classification model implemented for palliative care such as in [11]; the features and postures taken into account would be vastly different from those in the case of frameworks that might be applied for military applications, such as through-the-wall posture reconstruction frameworks in studies like [52]. In addition, the stakes for false detection and classification are likely to vary, so the effectiveness of the models would be affected accordingly.

### 4.1. Traditional/Statistical Learning

This subsection covers the literature on detection and classification that implements traditional/statistical learning methodologies. Traditional or statistical learning in ML encompasses various techniques that rely on statistical principles and algorithms to make predictions or decisions based on data. Some common types of traditional or statistical learning methods include the following:

- Linear Regression: A basic statistical technique used to model the relationship between a dependent variable and one or more independent variables by fitting a linear equation to observed data [112].
- Logistic Regression: Similar to linear regression but used for binary classification problems to predict the probability that an instance belongs to a particular class [113].
- Decision Trees: A non-parametric supervised learning method that partitions the data into subsets based on features, forming a tree-like structure where each internal node represents a decision based on a feature, and each leaf node represents a class label [114].
- kNN: A non-parametric lazy learning algorithm that classifies a data point based on the majority class of its *k*-nearest neighbors in the feature space [115].
- Naive Bayes: A probabilistic classifier based on Bayes’ theorem with the assumption of independence among features. It calculates the probability of a class given the input features [116].
- SVM: A supervised learning algorithm that constructs hyperplanes in a high-dimensional space to separate classes. It aims to find the hyperplane with the maximum margin between classes [117].
- PCA: A dimensionality reduction technique that transforms the data into a lower-dimensional space while preserving most of the variance. It is often used for feature extraction and data visualization [118].
- Cluster Analysis: Techniques such as K-means clustering or hierarchical clustering that group similar data points into clusters based on a distance metric [119].

These traditional learning methods provide foundational tools for solving various ML tasks, including regression, classification, clustering, and dimensionality reduction. However, they may have limitations in handling complex relationships in data compared to more advanced DL techniques that will be covered in the subsequent subsections.

Logistic regression and naive Bayes are some pedestrian learning methodologies employed in detection and classification frameworks. In [89], the two approaches were implemented to complement an FMCW-based sensing system for context-aware generic target detection using FFT input data. The study yielded a detection accuracy of 86.9%. Another instance of traditional learning as an augmentation for context-aware sensing was implemented in [120] for posture detection with the aim of fall-risk prevention in elderly subjects. Similar to the case in [71], the subjects’ body motions were recorded by UWB sensors, and signals are classified into standing, sitting, lying down, and idle. Location-specific classifiers significantly improved overall accuracy owing to context awareness being taken into account. The suggested decision tree classifier implemented 33 features from 10 s data segments to achieve an accuracy rate of 85%. Fall detection seamlessly combines posture and motion detection and classification for a variety of practical applications. Most notably, fall detection frameworks have become an integral component of elderly care. In scenarios with inadequate human labor to achieve this manually, RF-based (and indeed other sensing mechanisms) fall detection has been employed. Research into this topic can be cited in works such as [121], which emphasized the need for privacy and comfort in elderly care achieved through fall detection and prediction using wearable and non-wearable sensors. The paper introduced a novel pipeline for fall detection based on wearable accelerometer data via more than 7700 cross-disciplinary time series features from publicly available datasets. Feature reduction techniques, including mutual information and the Boruta algorithm, were applied, resulting in a set of 39 high-performing features being selected for classification. Various classical ML algorithms (random forest, logistic regression, SVM, naive Bayes, and decision trees) achieved high accuracy in fall detection for individual datasets using these high-performance features, with the pipeline’s generalization capability being validated across all three datasets, consistently demonstrating exceptional efficiency in fall detection. In [122], the study also tackled automatic, real-time posture detection and correction by using traditional learning, suggesting a method that makes use of three triaxial accelerometer and gyroscope sensors to detect and classify sitting and standing postures. Classification accuracy is increased via the use of multiple sensors, in contrast to one. By improving feature distinctiveness, PCA facilitates posture differentiation. In this case, there were four classifiers used: random forest, decision tree, multilayer perceptron, and SVM. Of these, RF exhibited the highest accuracy of 95.68% for nine different body postures (five sitting and four standing postures). The project is a prime example of the application of ubiquitous RF sensing via the use of wearable multisensor systems for posture and health monitoring. With a similar application scenario, the implementation of traditional learning for posture detection has been further discussed in [123]. The study presented a novel hybrid approach for posture detection that integrates different DL classifiers, namely, 1D-CNN, 2D-CNN, and LSTM, with traditional ML classifiers, namely SVM, logistic regression, and decision trees. The suggested hybrid approach seeks to improve overall performance by combining the predictive powers of DL and ML, with results from experiments conducted on a benchmark dataset showing an accuracy of more than 98%, demonstrating how well the hybrid approach works to improve posture detection accuracy.

kNN is also a commonly used traditional learning methodology, which is vital in scenarios where the underlying data distribution is not explicitly known and the decision boundaries are complex or non-linear [124]. Notable use cases for kNN classification include small-to-medium-sized datasets where computing distances between all pairs of data points remains computationally feasible, non-linear decision boundaries due to its ability to adapt to complex shapes of the decision boundary without explicitly modeling it, multiclass classification with multiple centroids, imbalanced datasets, and noise-sensitive classification use cases. With this in mind, it is no wonder that most context-aware fall detection sensing schema adopt kNN for classification. For instance, in [77], where, alongside SVM, kNN was used for fall and human activity detection, producing an accuracy ranging from 68.5% to 81%. These scenarios involve transition postures, thus making it difficult for classification/learning methodologies that would otherwise not be robust to noise or non-linear decision boundaries. Furthermore, in [26], the proposed gesture recognition framework employed kNN and random forest algorithm as a baseline comparison against the QAE algorithm implemented for feature extraction. Still, in the context of feature-based recognition, a naive Bayes classifier was used in addition to logistic regression in [89] to augment the latter’s accuracy.

Posture detection and classification frameworks also play an important role in gait rehabilitation and posture correction. An instance of such an application in this context is in [125], whereby, combined with robotics, the study investigated the integration of a computer vision system for stroke rehabilitation therapy. In this case, a rehabilitation robot is used to perform preprogrammed movements on healthy adults and stroke survivors. Multiclass posture classification relied on 3D trajectories of upper body joint positions recorded over time. Using time series data from the sensors, the study used an inertial measurement unit (IMU) sensor system to collect motion data. While shoulder elevation compensation was less accurately detected (AUC = 0.66, F1 = 0.07), the SVM classifier showed high accuracy in detecting lean forward compensation (AUC = 0.98, F1 = 0.82) and trunk rotation compensation (AUC = 0.77, F1 = 0.57). It is also possible to employ posture detection frameworks to evaluate the efficacy of rehabilitation treatments and differentiate between subjects with abnormal and normal gait patterns, such as in [126], via the use of ground reaction force (GRF) and PCA. The study’s justification is that individuals who have experienced severe physical trauma have a restricted gait range of motion, which continuously gets better with treatment. Consequently, gait and posture analysis are used to gauge how effectively the physiotherapy is working. The first two principal component coefficients (PCCs) are produced by PCA using time series GRF data from fracture patients (FG) and a control group (CG) both before and after treatment (FGB and FGA). According to the results, there was an elliptical area in the PCC plane that divides the CG from the FGB. FGA values also shifted in the direction of the CG region, suggesting that PCA can distinguish between different gait patterns and assess rehabilitation outcomes objectively. This illustrates the value of PCA, and indeed feature engineering in general, in measuring the efficacy of treatment from data and its effectiveness in gait analysis.

SVM has also been used in conjunction with more sophisticated DL methodologies in [127] to achieve accuracy values above 90% for real-time indoor posture detection. In context-aware RF sensing, SVM has played a role in human activity pattern recognition, with studies such as [50], whereby as many as eight activities were classified using an SVM-based approach. Further on activity recognition, ref. [39] presented a combined SVM and diffusion layer approach for classification in this context, yielding a 94.72% accuracy. Other frameworks that implement SVM could also be used for larger-scale motion detection in the event of search and rescue operations, as achieved in [81], with an accuracy of 96%.

The emphasis on posture classification has also implications for high-frame-rate applications like fall detection. In contrast, the algorithm proposed in [128] for smart home care system posture classification implemented a decision tree-based solution and focused on posture classification and fall detection by using ranges, or the distances between body parts, and worked with time series information. Furthermore, a posture window scheme that adapts to changes in the speed of posture change was suggested to guarantee real-time recognition. The method utilizes statistical data and accuracy as an evaluation metric. The outcomes show how effective the range-based algorithm is at classifying human posture and reliably and accurately detecting fall-down incidents. Upon comparison with a generic height-based algorithm, the proposed range-based algorithm outperformed the former in two out of the five postures considered, with the two algorithms performing similarly in the remaining three postures. This method has the potential to improve security and surveillance in smart home settings, especially in assisted living and medical settings. In [78], where another fall detection scheme was proposed, an SVM classifier was used with fused feature inputs, yielding a test accuracy of 99.77%. Traditional learning can also be implemented via PCA for feature extraction [71] or as a prelude to classification, as used in [39], before SVM was used for classification.

Another example of a cutting-edge computer vision-based fall detection system designed for senior citizen home care monitoring can be cited in [129], whereby the method extracted the human body posture in the foreground by using advanced post-processing techniques, mainly background subtraction. Evaluation metrics included the detection accuracy and false positive rates. A directed acyclic graph support vector machine was subsequently applied for posture recognition, and the training strategy, in this case, involved a dataset with labeled postures. Ellipse fitting and projection histogram information were then utilized for posture differentiation. Posture classification is made possible by these features, which are inputs for a directed acyclic graph support vector machine. The results were then combined with data obtained from the floor to determine fall incidents. The system’s effectiveness in protecting senior users’ well-being was demonstrated by the achieved 97.08% successful fall detection rate and low 0.8% false detection rate in a simulated home setting according to an evaluation conducted on a dataset of 15 subjects.

Feature extraction is an additional aspect that has contributed massively to the improvement of computationally light solutions for radar-based frameworks, as examined in [130], where the use of DL was implemented for extracting features for SAR target classification. According to the survey, feature engineering is key to circumventing the curse of dimensionality that may hinder the efficacy of otherwise real-time detection and classification solutions. Feature extraction is also notably pertinent in classification via segmentation, as seen in [131] where it was implemented to improve the diagnostic value of ultrasound liver images by automatically classifying liver images into four groups: benign, malignant, cystic, and normal masses. Texture features are essential for classification and can be extracted using spectral and statistical methods. To select features as effectively as possible and help determine which features are the most discriminative, PCA is utilized. Specifically, grey-level co-occurrence matrix, gray-level run-length matrices, energy laws, and Gabor Mean feature extraction methods were implemented to reduce the dimensionality of the data from 33 to 7, 9 to 3, 22 to 5, and 24 to 5, respectively. After this, K-means clustering and neural network-based automatic classifiers were used to classify the scanned images into four categories: Normal, Metastases, Benign, and Cyst. The analysis of results presented depicts an improvement in the execution time with the application of PCA, despite there being no or negligible change in the classification accuracy. However, the authors purport that the latter could be improved by employing further preprocessing techniques to eliminate noise and improve the contrast, thus improving the quality of the images. Ref. [130] is in line with the current medical ultrasound imaging trend of automating diagnostic procedures. Through the integration of PCA and neural networks, the study addressed a crucial component of diagnostic applications in abdominal organ imaging and added to the ongoing efforts to increase the efficiency and accuracy of liver lesion classification. Another facet that could be explored would entail the difficulties in developing firmware and intelligent decision-making software for wearable motion-sensing devices used for posture monitoring and fall detection, as in [132]. In addition to introducing the k-means clustering algorithm to semi-automatically extract training examples from motion data, it offers a flexible framework for developing firmware. The study used neural networks and softmax regression combined with one- and two-level classification networks that are trained offline using the suggested technique. The two-level network’s cross-validation yielded 100% accuracy for both fall events and non-fall activities. Data collection was accomplished via devices assembled with commercially available components and programmed with the suggested framework. Motion detection also benefits from feature engineering for more streamlined solutions. One such scenario is in [133], which addressed data calibration neglect by proposing a Human Motion Target Posture Detection Algorithm within the IoT framework, which gathers IoT-based human motion target images and uses the eight-star model for feature extraction via semi-supervised learning. Multifeature fusion is used to create the 17-dimensional feature vectors. Efficient classification of image blocks is achieved by random fern classifiers that have been optimized through semi-supervised learning training. Random fern classifiers fall under the category of traditional or statistical ML methods. They are a type of ensemble learning technique that combines multiple weak classifiers, known as ferns or decision trees, to make predictions. The suggested algorithm showed its accuracy and efficiency in human motion posture detection within the IoT environment, with a 92.5% correct classification rate for various poses, an average F value of 0.95 for attitude detection, and short computation times.

### 4.2. Convolutional Neural Networks

Fall detection systems can also utilize UWB radar sensors to achieve posture recognition, as employed in [71] to ensure the safety of senior users while preserving their privacy. Human body gestures are recorded by UWB sensors, and signals are classified into standing, sitting, lying down, and idle categories using a CNN. The evaluation metrics that follow are sensitivity and accuracy. As such, the model makes use of raw data. The CNN model demonstrated its effectiveness in recognizing static human postures in private settings with an astounding 93% accuracy rate. In [42], the study presented a novel approach for classifying digits written in mid-air using hand gestures captured by impulse UWB radar sensors. Unlike conventional methods that rely on raw data matrices or feature groups for classification, the proposed method leverages the hand’s mid-air trajectory for robust classification, with the approach involving three stages: signal preprocessing, hand motion localization, and trajectory transformation into an image for classification using a CNN. By focusing on trajectory-based classification, the method achieved robustness to variations in orientation, distance, and hand shape and size, with an accuracy of 92%. The proposed system offers a user-friendly and accurate mid-air handwriting modality without requiring extensive training databases, making it suitable for real-world applications. The proposed system in [76] transforms mmWave signals into point clouds and employs denoising, enhanced voxelization, data augmentation, and dual-view ML for accurate and efficient recognition. Specifically, the method leverages spatial–temporal point clouds and the symmetry properties of radar rotations and utilizes a dual-view CNN for learning. Evaluation on a dataset comprising seven activities demonstrated impressive performance, with accuracies of 97.61% for fall detection and 98% for activity classification, outperforming conventional ML schemes. CNNs also contribute to HAR classification where noise sensitivity is required, as pursued in [72], where the proposed approach utilizes a CWT for signal decomposition and a two-channel CNN for high-level feature learning from time-domain signals and CWT spectrograms. Denoising techniques such as Savitzky–Golay filtering were applied to the I/Q signals, and both noisy, and denoised signals were used for DL data augmentation. Experimental results show an overall classification accuracy rate of 98.85% for a two-branch CNN architecture and 95.3% for a one-branch 2D-CNN, highlighting the efficacy of the dual-channel CNN model in enhancing human motion recognition and classification capabilities using radar signals. A vehicle speed detection approach that uses smartphones’ acoustic sensors was explored in [63]. The proposed hybrid detection system for vehicle speed (HDSpeed) leverages a combination of CNNs and LSTM networks to separately model the acoustic patterns of electric vehicles (EVs) and gasoline vehicles (GVs). By extracting distinct features from the acoustic signals of EVs and GVs, HDSpeed achieves accurate speed estimation with an average error of 2.17 km/h in real driving environments. Additionally, the method employs active acoustic sensing to calculate fine-grained speed by detecting distance changes between the smartphone and passing vehicles while effectively eliminating interference from surrounding moving objects. Another example of CNN implementation for through-the-wall sensing is presented in [36] which explored an open-source dataset that highlights the use of active RF sensing for non-contact human information detection and monitoring. The dataset recorded human motion statuses, and a CNN-based approach for human motion status recognition was suggested, which yielded a recognition accuracy of more than 99.7% for three motion statuses. The robustness of CNN to multifeature datasets also comes into play in studies like [51], which implemented a CNN model with eight complex human activities, namely, hug, pat, push, kick, high-five, handshake, bow, and boxing. Such a framework can be instrumental in monitoring abuse in child and palliative care. Having cited studies such as [52,53] that deal with research into through-the-wall RF sensing, the subsequent part of these frameworks usually involves posture detection and classification. Such a scenario was explored in [134]. Inspired by cross-modal learning, the proposed method in this study applies a supervision prediction learning approach using a CNN for through-the-wall posture reconstruction while dealing with the problem of low spatial resolution. The training dataset was created by simultaneously gathering optical images and TWRI signals with the help of a radar and an optical camera. During training, the CNN is guided to predict human skeletons from TWRI signals by computer-vision-based supervision, which creates ground-truth skeletons from optical images. The model exhibited complete predictions in wall-occlusive scenarios using only TWRI signals. Comparison tests demonstrate accurate qualitative outcomes with wall occlusion and quantitative parity with vision-based approaches in non-occlusive scenarios. By focusing on CNN-based methods, the study advances human posture reconstruction in TWRI for military scenarios that may require stealth sensing. Although most of the previous approaches have mostly focused on identifying individuals in standing positions, ref. [24] tackles the difficulties caused by the variety of human postures, including standing, sitting, lying, and crunching, particularly from various angles. The study treats posture classification as a multiclass detection problem and presents an unsupervised method to identify characteristic human postures. The suggested approach, which makes use of the YOLO CNN, achieved good outcomes from six perspectives with relatively high recall and precision (99%). The collaboration of ML/AI and human posture classification is an additional area that has attracted immense research interest. As explained extensively in surveys such as [135], for a radar-based framework, ML can be introduced at the radar signal processing stage or at the posture detection stage, as in [127] for instance, where the study aimed for real-time posture detection for standing, sitting, and walking indoor scenarios. It employed vision sensors for data acquisition. Using a lightweight deep CNN (DCNN) called MobileNet, the study highlights higher recognition rates using fewer parameters. Techniques that improved generalization and lower hyperparameters included regularization, transfer learning (TL), and neural architecture search. Furthermore, the study employed methods like TL, DA, and SVM models, outperforming more sophisticated counterparts like ResNet50V2, InceptionV3, and DenseNet201. With a computational time of 3974 s, the suggested MobileNet-SVM hybrid achieved optimal performance, exhibiting 92.12% accuracy, 95% AUC, 92% recall, and 93% precision. This method significantly improves posture classification techniques by creatively capturing temporal and depth characteristics.

### 4.3. Time Series/Domain with Deep Learning

Some types of learning methods in ML that use time series/domain DL include the following:

- Recurrent Neural Networks (RNNs): RNNs are designed to work with sequences of data and are well suited for time series analysis. They can capture temporal dependencies in the data by maintaining internal memory [136].
- LSTM Networks: LSTMs are a type of RNN that can capture long-term dependencies in time series data. They are particularly effective at learning patterns in sequences with long time lags [137].
- Gated Recurrent Units (GRUs): GRUs are another type of RNN that is similar to LSTMs but has a simpler architecture. They are often used in scenarios where computational resources are limited [138].
- Temporal Convolutional Networks (TCNs): TCNs apply convolutional operations to time series data, allowing them to capture local patterns at different temporal scales. They are known for their parallel processing capabilities and have been shown to perform well in sequence modeling tasks [139].
- Transformer-Based Models: Transformer architectures, originally developed for natural language processing, have also been adapted for time series data. These models use self-attention mechanisms to capture long-range dependencies in sequences, making them suitable for time series forecasting and classification tasks [140].

Robust motion and posture detection for the elderly can also take the form of sit-to-stand and stand-to-sit sensing via the use of inertial sensors mounted on the subjects’ trunk, as seen in [141]. The method uses dynamic time warping (DTW) to classify trunk acceleration patterns according to how well they match predefined templates. DTW is a dynamic programming technique used to measure the similarity between two temporal sequences that may vary in length or speed. It is often employed in time series analysis, signal processing, and pattern recognition tasks [142]. Using real-world data from patients with chronic pain and healthy subjects, the classifier was validated. Compared to generic templates, custom templates produced the highest accuracy (over 95% for healthy subjects and 89% for chronic pain). The study notes that while predictions from controlled conditions are considered excessively optimistic, real-world tests are crucial. Furthermore, the suggested wearable system shows promise in clinical assessment, identifying differences in postural transitions between healthy, chronic pain, and frail older subjects. Different proposed solutions also implement different posture estimation and classification approaches. For instance, in [143] the study suggests a fall detection model that uses a machine vision framework in conjunction with the OpenPose human posture estimation algorithm. The sensor modality involves vision-based methods for human posture estimation; thus, the likely data domain adopted is the time series, capturing the dynamics of human postures. The evaluation metrics consequently include detection and classification accuracy. By removing non-human key points, integrating SSD-MobileNet object detection with OpenPose improves accuracy, lowers false detection, and strengthens the algorithm’s robustness in dynamic environments. The technique uses the support vector data description (SVDD) classification algorithm to extract features from human joint points and shows accuracy rates exceeding 93% in fall detection. In other studies, different learning methods were compared and contrasted using the same experimental setup and data. In this regard, the study presented in [125] yielded results upon implementing an SVM classifier that are then mirrored when an RNN classifier takes temporal dependencies into account. However, the detection accuracy was generally lower in stroke survivors, indicating that to improve the accuracy of compensatory motion detection during stroke rehabilitation, improvements should be made to the definition of motion ranges, camera placement, exercise intensity, seat height adjustment, and comprehensive session recording. As explored in [44,70], context awareness is an invaluable addition to the motion prediction pipeline as it improves the learning process by streamlining the information required by the model to produce an architectural template for detection and classification. In [144], existing RNN implementations have been further improved to this end. To model time-dependent representations for short-term motion prediction and long-term motion synthesis, the study evaluated various RNN architectures, loss functions, and training procedures, and it proposed three modifications to standard RNN models to enhance performance, namely, sequence-to-sequence architecture with sampling-based loss—which involves an encoder that receives the inputs and generates an internal representation—and a decoder network that takes the internal state and produces a maximum likelihood estimate for prediction, residual architecture, and multiaction models where a single model is trained to predict multiple actions, in contrast to existing works, such as explored in [145,146]. On the other hand, with data collected in the time series domain via a wearable sensor system with three tri-axial accelerometers [147], the paper focuses on sitting and standing postures, suggesting a novel DL model to classify 9 different postures. This model combines a fully convolutional network (FCN) with an LSTM. The combined FCN-LSTM network achieved the highest average classification accuracy at 99.91%, indicating superior performance for real-time posture correction according to the results. In comparison, the accuracy of individual FCN and LSTM networks was 97.88% and 88.47%, respectively. The models’ complexity and execution time are also assessed. Despite not implementing RF sensing and the sensing mode, this study still provides invaluable insight into feature selection and subsequent performance metrics in terms of computational complexity, execution, and accuracy trends over time. Furthermore, motion detection plays a part in in-vehicle driver behavior analysis for accident prevention. Ref. [148] highlights this and tackles the identification of driver mental fatigue in intelligent vehicles. The work presented an innovative approach to tracking head posture motions using XSENS motion capture tested on a driver-in-loop simulator. The method used 3D time series head angular acceleration data to train and test a newly modified bi-directional long short-term memory (BiLSTM) deep neural network with a rectified linear unit layer. The classifier achieved an overall training accuracy of 99.2%, sensitivity of 97.54%, precision, and F1 scores of 97.38% and 97.46%, respectively. These results show the classifier’s superior performance over current methods and conventional ML tools. The study further offers recommendations for future research directions in this field as well as an in-depth examination of its limitations. Some existing studies have also performed comparisons in the context of robust activity recognition with a myriad of DL techniques, as in [149], which presents a robust multilabel HAR framework designed for ambient assisted living (AAL) systems that consists of two main modules: an activity classification module and a classification error detection and correction module. The activity classification module employs various ML models, namely, Markov and Bayesian network models, for modeling relationships between variables, followed by naive Bayes, decision tree, SVM, and random forest, as well as a deep residual bi-directional LSTM (Deep-Res-Bidir-LSTM) network to predict human activities, while the error detection and correction module utilize acyclic directed graphical models to identify and rectify classification errors. Evaluated on the Opportunity dataset, the proposed framework demonstrated enhanced performance in HAR by effectively detecting and correcting classification errors. The RFs model yielded the highest average F1 measure, 81.90%, while for Deep-Res-Bidir-LSTM, the average F1 measure was 81.13%. Future research directions may include further refinement of the error detection and correction mechanisms, exploration of real-world deployment scenarios, and adaptation to diverse datasets and activity contexts within AAL systems.

### 4.4. Advanced Learning

Some types of learning methods in ML that use advanced learning techniques include the following:

- Generative Adversarial Networks (GANs): GANs consist of two neural networks, a generator and a discriminator, which are trained simultaneously in a competitive manner. GANs are widely used for generating synthetic data, data augmentation, and unsupervised learning tasks [150].
- Autoencoders: Autoencoders are neural networks trained to reconstruct their input data at the output layer. They are commonly used for feature learning, dimensionality reduction, and anomaly detection [151].
- VAEs: VAEs are a type of autoencoder that learns a probabilistic distribution over the input data. They are used for generating new data samples and performing tasks such as image synthesis and data generation [152].
- Reinforcement Learning (RL): RL is a type of learning where an agent learns to interact with an environment to achieve a goal by maximizing a cumulative reward signal. RL algorithms are used in various applications such as game playing, robotics, and autonomous systems [153].
- Transfer Learning: Transfer learning involves transferring knowledge from one task to another related task. Pretrained models are fine-tuned on new datasets to solve different tasks, reducing the need for large amounts of labeled data [154].
- Meta-Learning: Meta-learning, or learning to learn, involves training models on multiple tasks to learn generalizable knowledge or meta-knowledge. Meta-learning algorithms are used for few-shot learning, adaptation to new tasks, and continual learning [155].
- Self-Supervised Learning: Self-supervised learning is a type of unsupervised learning where models are trained to predict certain properties of the input data. Self-supervised learning methods are used for pretraining models on large unlabeled datasets, which can then be fine-tuned on smaller labeled datasets for downstream tasks [156].

A neural fuzzy network (NFN) falls under the category of hybrid learning methods, which combine elements of both traditional or statistical learning and advanced learning. It combines the principles of fuzzy logic, which deals with uncertainty and imprecision in data, with neural networks, which are powerful tools for learning complex patterns and relationships in data. Implementing NFNs, there exist frameworks that are based on the classification of predefined postures, such as those exhibited in [152], which presented a novel approach to human body posture classification using an NFN with an emphasis on emergency detection resulting from accidental falls. In this case, the four primary postures that the system distinguished between were standing, bending, sitting, and lying (presumably on the floor/ground), with posture classification being achieved by segmenting the human body silhouette and extracting features using silhouette projection and a discrete Fourier transform applied to histograms. The silhouette length-width ratio and significant Fourier transform coefficients are two of these features that are fed into the NFN classifier. Experiments validate that the suggested SONFIN approach is highly accurate, recognizing the four postures with an average recognition rate of 97.8%. This makes it useful for home care emergency detection, particularly for users with posture- and fall-associated disorders. NFNs are also instrumental in object motion detection and classification, as explored in [157]. The paper investigates the application of an adaptive neuro-fuzzy inference system (ANFIS) for classifying moving objects in street surveillance scenarios. The research focuses on three key processes: object detection, discriminative feature extraction, and target classification, with a specific emphasis on pedestrian, motorcyclist, and car detection. The ANFIS model is designed with three output parameters, each incorporating three inputs and 27 Sugeno rules. Extensive experimentation was conducted on significant features across three street scene datasets with varying background complexities. The evaluation performance analysis demonstrates that the proposed approach achieved a classification accuracy of 93.1% for street scenes with moving objects, outperforming solely neural network or fuzzy-based approaches. The paper highlights the potential of neuro-fuzzy systems for improving object classification accuracy in surveillance applications and suggests avenues for future research, such as optimizing the model architecture and exploring additional feature extraction techniques to further enhance performance. Advanced learning is also implemented via RL in studies such as [158]. In this scenario, the IVADC-FDRL model was introduced, which leverages deep RL (DRL) techniques for anomaly detection and classification in intelligent video surveillance applications. The model consists of two main stages: anomaly detection and classification. Anomaly detection is performed using the faster RCNN model with a residual network as a baseline, which detects anomalies as objects. Subsequently, deep Q-learning (DQL)-based DRL is employed for anomaly classification. Extensive experimentation on the UCSD anomaly dataset validated the effective performance of the IVADC-FDRL model, achieving maximum accuracies of 98.50% and 94.80% on Test004 and Test007 datasets, respectively. The study highlights the potential of integrating DRL techniques with object detection models for robust anomaly detection and classification in video surveillance systems. Future research directions may include further refining the IVADC-FDRL model, exploring real-world deployment scenarios, and addressing challenges related to scalability and computational efficiency.

## 5. Challenges to RF Sensing

The deployment of RF sensing in the various heretofore-explored use cases is a testament to its efficiency and highlights its main advantages. However, the successful implementation of RF sensing is restricted by some challenges and limitations that must be resolved to optimize its potential. In this section, we articulate some common challenges facing RF sensing and outline their proposed solutions, as proposed in the existing literature. The succeeding subsections summarize the key FL challenges and the associated solutions in the existing literature.

### 5.1. Signal Processing

Signal processing plays a crucial role in RF sensing, but several issues can impact its effectiveness. Some common signal processing issues, along with their corresponding impacts and proposed solutions, include the following.

#### 5.1.1. Noise

Noise is an inherent part of RF signals and can originate from various sources, including thermal noise, environmental factors, and electromagnetic interference. Noise can degrade the quality of received signals and affect the accuracy of detection and classification.

Impact: High levels of noise can obscure the signal of interest, making it challenging to detect and extract relevant information.

Solution: Employing noise reduction techniques such as filtering, averaging, and adaptive thresholding [159] can help mitigate the impact of noise on RF sensing systems. Advanced algorithms, including Kalman filters [160] and wavelet denoising using ANNs as studied in [161], can further enhance signal-to-noise ratio and improve detection performance.

#### 5.1.2. Clutter

Clutter refers to unwanted echoes or reflections from static objects in the environment, such as buildings, vegetation, or terrain. Clutter can interfere with the detection of targets and lead to false alarms or missed detections.

Impact: Clutter can obscure genuine targets and reduce the accuracy and reliability of RF sensing systems.

Solution: Implementing clutter rejection algorithms, such as those explored in [162] that distinguish between clutter and legitimate targets based on characteristics like motion, size, or Doppler shift, can help mitigate clutter-related issues. Techniques like adaptive thresholding and pulse compression can also improve clutter suppression capabilities.

#### 5.1.3. Multipath Propagation

Multipath propagation occurs when RF signals take multiple paths to reach the receiver, leading to signal reflections and ghosting effects. Multipath propagation can distort received signals and complicate target detection and localization.

Impact: Multipath propagation can cause signal distortion, ghost targets, and inaccuracies in ranging and positioning.

Solution: Employing techniques such as beamforming [163], diversity reception, and equalization can mitigate the effects of multipath propagation. Advanced algorithms, including maximum likelihood estimation and least squares estimation, can help estimate and compensate for multipath-induced distortions, improving the accuracy of signal processing.

#### 5.1.4. Interference

Interference from external sources, such as other RF devices, electronic noise, or intentional jamming, can disrupt signal transmission and reception. Interference can degrade signal quality and impair the performance of RF sensing systems.

Impact: Interference can lead to false alarms, missed detections, and overall degradation of system performance.

Solution: Implementing interference mitigation techniques such as frequency hopping, as in [164] to combat intentional interference, spread spectrum modulation [165], and adaptive filtering [60], can help suppress interference signals and improve signal integrity. Dynamic spectrum access and cognitive radio techniques can also enable adaptive and intelligent responses to changing interference conditions, enhancing system robustness.

### 5.2. Range and Resolution

Range and resolution are critical factors in RF sensing that can significantly impact its effectiveness. Some common range and resolution issues, along with their corresponding impacts and proposed solutions, include what follows.

#### 5.2.1. Limited Range

Limited range refers to the maximum distance over which RF signals can be reliably detected and measured. The limited range can restrict the coverage area of the sensing system and limit its applicability in scenarios requiring long-distance sensing.

Impact: Limited range can result in incomplete coverage of the sensing area, missed detections of targets beyond the range limit, and reduced overall system effectiveness.

Solution: Increasing the transmit power of the RF signals, optimizing antenna configurations as in [166], and employing signal amplification techniques [167] can help extend the range of RF sensing systems. Additionally, utilizing advanced signal processing algorithms, such as coherent integration used in [168] to combat Doppler ambiguity caused by range limitations and pulse compression as in [169] to reduce waveguide length and improve efficiency, can improve the system’s ability to detect weak signals at longer distances.

#### 5.2.2. Spatial Resolution

Spatial resolution refers to the ability of an RF sensing system to distinguish between closely spaced targets or features in the sensing environment. Spatial resolution is crucial for accurately identifying and localizing targets, particularly in dense or cluttered environments.

Impact: Inadequate spatial resolution can lead to poor target localization, false alarms, and reduced overall detection accuracy, especially in scenarios with closely spaced objects.

Solution: Increasing the number of receiving antennas, implementing advanced beamforming techniques [170], and employing higher-frequency RF signals can enhance spatial resolution in RF sensing systems. Additionally, using sophisticated signal processing algorithms, such as super-resolution techniques and adaptive filtering, can improve the system’s ability to resolve fine details and discriminate between closely spaced targets.

#### 5.2.3. Ambiguity

Ambiguity in RF sensing refers to the inability to accurately determine the distance or location of targets due to overlapping or indistinct signal characteristics. Ambiguity can arise from factors such as signal reflections, multipath propagation, and interference.

Impact: Ambiguity can lead to inaccuracies in target localization, erroneous distance measurements, and misinterpretation of sensor data, compromising the reliability and effectiveness of the sensing system.

Solution: Implementing range gating techniques, frequency modulation methods such as binary-phase shift keying and frequency shift keying [171], and signal processing algorithms such as pulse compression [169] can help mitigate ambiguity in RF sensing systems. Additionally, utilizing advanced radar waveforms, such as FMCW signals, can improve range resolution and reduce the effects of ambiguity.

### 5.3. Sensitivity and Detection

Sensitivity and detection are critical aspects of RF sensing that can significantly impact the system’s performance. Some common sensitivity and detection issues, along with their corresponding impacts and proposed solutions, include what follows.

#### 5.3.1. Low Sensitivity

Low sensitivity refers to the inability of an RF sensing system to detect weak or low-power signals from targets. Low sensitivity can result from factors such as signal attenuation, noise interference, and inadequate receiver sensitivity.

Impact: Low sensitivity can lead to missed detections of faint or distant targets, reduced detection range, and decreased overall system reliability and effectiveness.

Solution: Improving receiver sensitivity by using low-noise amplifiers, optimizing antenna design and placement, and minimizing signal losses in the RF chain can enhance the sensitivity of RF sensing systems. Additionally, employing signal processing techniques such as coherent integration, as in [172]—whereby this was implemented to improve the detection of a weak maneuvering airborne target—and matched filtering, as in [173]—where this was implemented to enhance heart rate detection using a Doppler radar-based system—and noise reduction algorithms can help improve the system’s ability to detect weak signals in noisy environments.

#### 5.3.2. Signal Interference

Signal interference occurs when unwanted signals from external sources, such as electromagnetic interference (EMI) or co-channel interference, disrupt the detection process. Signal interference can degrade the quality of the received signal and interfere with target detection.

Impact: Signal interference can result in false alarms, a degraded signal-to-noise ratio (SNR), and decreased detection accuracy, leading to misinterpretation of sensor data and reduced system performance.

Solution: Implementing interference mitigation techniques such as frequency hopping [164], spatial filtering explored in [174], and adaptive signal processing algorithms [175] can help mitigate the effects of signal interference in RF sensing systems. Additionally, utilizing frequency-selective filters, shielding techniques, and frequency diversity schemes can reduce the impact of external interference on signal detection.

#### 5.3.3. Detection Threshold

The detection threshold refers to the minimum signal strength or amplitude required for the RF sensing system to detect a target. The detection threshold directly affects the system’s ability to detect and discriminate between targets of varying sizes and distances.

Impact: Inappropriate detection threshold settings can lead to missed detections of small or low-power targets, increased false alarm rates, and reduced overall detection sensitivity.

Solution: Adjusting the detection threshold based on environmental conditions, target characteristics, and system requirements can optimize the performance of RF sensing systems. Fine-tuning detection algorithms, setting adaptive threshold levels [159], and employing dynamic thresholding techniques can help optimize detection sensitivity while minimizing false alarms and maximizing target detection capabilities.

### 5.4. Real-Time Processing

Real-time processing is essential in RF sensing applications, but several challenges can affect its effectiveness. Some common real-time processing issues, along with their corresponding impacts and proposed solutions, include what follows.

#### 5.4.1. Latency

Latency refers to the delay between signal acquisition and processing, which can impact the responsiveness of the system. Latency can result in delayed detection or classification of targets and reduce the system’s ability to provide timely information.

Impact: High latency can lead to missed events, degraded performance in dynamic environments, and reduced overall system efficiency.

Solution: Implementing efficient algorithms and optimized processing pipelines can help minimize latency in RF sensing systems. Techniques such as parallel processing and hardware acceleration expounded in [176], as well as algorithmic optimization for classification use cases already implemented in studies such as [177], can reduce processing time and improve real-time responsiveness.

#### 5.4.2. Computational Complexity

The computational complexity of signal processing algorithms can impact real-time performance, especially in resource-constrained environments. Computational complexity refers to the amount of computational resources required to execute a particular algorithm within a specified time frame.

Impact: High computational complexity can lead to increased processing time, resource utilization, and power consumption, limiting the feasibility of real-time processing.

Solution: Employing lightweight algorithms, algorithmic simplification, and hardware optimization techniques can help reduce computational complexity and improve real-time performance. Techniques such as pruning explored in [178], adapting quantization as implemented in [179] for learning in detection and classification, and model compression [180], can also streamline algorithm execution without compromising accuracy.

#### 5.4.3. Data Throughput

Data throughput refers to the rate at which data are processed and transmitted within the system. Data throughput constraints can arise due to limited bandwidth, memory capacity, or processing resources, affecting the system’s ability to handle high volumes of data in real-time.

Impact: Insufficient data throughput can lead to data loss, buffer overflow, and degraded system performance, particularly in high-speed or high-density sensing scenarios.

Solution: Optimizing data transmission protocols, implementing data compression techniques [181], and leveraging parallel processing architectures, such as in [176], can help increase data throughput and improve real-time processing capabilities. Hardware acceleration, such as the use of graphics processing units (GPUs) [182] or field-programmable gate arrays (FPGAs), can also enhance data processing speed and efficiency.

### 5.5. Cross-Context Adaptation of Model-Driven Solutions

Consider the following case: there exists an arbitrary context-aware RF-based sensing framework for detection and classification. This framework, which could implement active (FMCW) or passive sensing (WiFi CSI), is accompanied by a feature engineering schema for the extraction of important features in the data, as well as gives rise to new synthetic features and then implements an arbitrary DL model for classification. This framework is inherently model-driven. In an attempt to adapt this same framework for the same purpose, albeit in a different context, some challenges are bound to be faced.

#### 5.5.1. Compatibility Between Sensing/Data Acquisition Methods

Impact: Different sensing methods, such as active (FMCW) and passive (WiFi CSI), may have different data formats, sampling rates, and noise characteristics, making it challenging to integrate them into a unified framework.

Solution: Develop data preprocessing techniques to standardize the data formats and mitigate differences in sampling rates and noise levels. Additionally, explores data fusion approaches to effectively combine information from multiple sensing modalities while accounting for their inherent differences.

#### 5.5.2. Compatibility Between the Implemented DL Model and the Data

Impact: The DL model may be designed with specific assumptions about the input data format, distribution, and characteristics. If the data collected from different sensing methods do not align with these assumptions, they may lead to suboptimal model performance or even failure.

Solution: Conduct thorough data analysis and preprocessing to ensure that the input data meets the requirements of the DL model. Additionally, consider model adaptation techniques such as transfer learning [154] or domain adaptation to fine-tune the pre-trained DL model on the target data domain.

#### 5.5.3. Signal Processing Inconsistencies Across Different Sensing Modalities

Impact: Each sensing modality may require specific signal processing techniques for data cleaning, feature extraction, and noise reduction. Incompatibilities in signal processing methods can lead to inconsistencies in the quality and reliability of the extracted features, affecting the overall classification performance.

Proposed Solution: Develop adaptive signal processing algorithms that can automatically adjust parameters and processing steps based on the characteristics of the input data. Additionally, investigates domain-specific signal processing techniques tailored to each sensing modality to ensure optimal feature extraction and classification accuracy.

### 5.6. Privacy-Preserving Mechanisms and Protocols for Context-Aware RF Sensing Systems

As RF sensing systems become more widespread in real-world applications, concerns about privacy related to sensitive RF data have become a significant issue. Context-aware RF sensing often requires collecting data about human behaviors, poses, and environments, highlighting the need for privacy-preserving methods to protect user information while maintaining high detection and classification accuracy. This discussion will cover three main approaches: data anonymization techniques, federated learning, and selective data capture.

#### 5.6.1. Data Anonymisation Techniques

Data anonymization is an important privacy-preserving technology that ensures personally identifiable information (PII) or sensitive user data cannot be linked back to specific individuals. In the context of RF sensing, anonymization aims to ensure that features acquired from RF signals do not betray human identities while allowing for effective detection and classification:

- Signal Processing for Anonymization: Techniques like noise addition, data perturbation, and data obfuscation can disguise identifying features in raw RF signals without significantly reducing their classification utility.
- K-Anonymity and Differential Privacy: K-anonymity ensures that each participant in a dataset is indistinguishable from at least K-1 others, whereas differential privacy requires introducing controlled noise into the data to hide individual contributions. These technologies are especially beneficial for exchanging RF sensor datasets across multiple applications or organizations.
- Feature-Level Anonymization: Specific features like gait patterns or breathing rates can be retrieved and anonymized using feature transformation or encoding techniques to reduce privacy risks while maintaining task-relevant data.

#### 5.6.2. Federated Learning

Federated learning (FL) is a decentralized machine learning approach that enables RF sensing systems to train models together without exchanging raw data. Instead, only model changes are communicated, dramatically lowering the danger of data breaches while increasing privacy.

- Local Training and Model Aggregation: In an FL configuration, each device trains a local model with its own RF data and only shares model parameters (e.g., gradients) with a central server. The server combines these updates to form a global model, keeping raw data local to the device.
- Secure Aggregation: To improve privacy, secure multiparty computation (SMPC) and homomorphic encryption can aggregate model updates without revealing individual contributions, preserving sensitive RF data while training.
- Privacy versus Accuracy Tradeoffs: Federated learning allows for high detection accuracy while protecting privacy. Issues such as non-IID (non-independent and identically distributed) data and communication overheads must be addressed to optimize performance in resource-constrained IoT contexts.

#### 5.6.3. Selective Data Capture

Selective data capture is a deliberate strategy of minimizing the amount of data acquired by RF sensing systems, hence decreasing privacy hazards and focusing on task-relevant information:

- Context-Driven Capture: By implementing context awareness, RF sensing systems can capture data only when certain circumstances are met (for example, detecting motion or posture changes). This reduces the acquisition of unneeded or irrelevant data, which could jeopardize privacy.
- Edge Processing and Prefiltration: Processing RF data locally on edge devices enables real-time feature extraction and filtering before transmission. Only high-level features or summaries are published, which reduces the possibility of disclosing raw, sensitive data.
- Privacy-Preserving Triggers: Sensors can be programmed to capture data only when predefined thresholds or patterns are identified, such as anomalous behavior or occurrences that require monitoring.

## 6. Future Research Directions

RF sensing for object, posture, and motion detection, as well as classification, is a burgeoning field with several promising future directions. This technology has far-reaching applications in healthcare, robotics, smart homes, and beyond. Several key areas are expected to shape the future of RF-based posture and motion detection and classification:

1. Enhanced Resolution and Accuracy: Future research will focus on improving RF resolution and accuracy in detecting and classifying human postures and motions. Advanced signal processing techniques, higher-frequency RF systems, and more sophisticated algorithms will contribute to finer-grained detection and classification. Loosely attached to this point, the issue of seamless frame-wise detection and classification could be highlighted. As pointed out in [24], quick transitions in real-time motion hurt classification in that it may be quite difficult to decide on the perfect balance between the number of features to consider to achieve satisfactory classification accuracy within a reasonable computational time threshold.
2. Multimodal Sensor Fusion: Subsequent investigations will concentrate on enhancing RF resolution and precision in identifying and classifying human positions and movements. This calls for multiple sensor modalities to work in collaboration with RF, as seen in [141]. Finer-grained detection and classification will be facilitated by more sophisticated algorithms, higher frequency RF systems, and advanced signal processing techniques.
3. Real-time and Edge Processing: It is critical to have real-time, on-device RF data processing capabilities at the edge. It makes systems more responsive and lowers latency, which makes them better suited for applications like robotics, security, and fall detection.
4. ML and AI: Using AI and ML to analyze RF data will improve the accuracy of posture and motion detection and classification, as cited in [123,127]. DL algorithms can enhance system performance and adjust to different situations. A key element to consider in this regard would be to focus on reducing the computational workload of the ML model. Feature engineering techniques concentrated on compacting the number of features needed to train the model would be one of the most important topics to take into consideration in this regard, as has already been spearheaded in existing research works like [131,133]. To this effect, the augmentation of VAEs, PCA, or t-SNE in the preprocessing phase of the model would be of much help.
5. Large Language Models (LLMs) and Context-Aware Sensing: Discussing LLMs as context-aware models (in their current application domain), the following features can be gleaned: the comprehension and generation of contextual information; multimodal data generation; adaptive learning with real-time adaptation and personalization; predictive capabilities concerning anticipating user needs and scenario analysis; and scalability and generalization with large-scale training and transfer learning. With these in mind, the future of incorporating LLMs in context-aware sensing and learning is promising, with the potential to revolutionize how intelligent systems interact with and adapt to their environments. Through the fine-tuning of cross-context model-driven solutions (in a more abstract sense), the workability of LLMs and existing and future LLM-based solutions in the domain of context-aware sensing can be realized.
6. Privacy-Preserving Solutions: Privacy concerns are becoming more prevalent as RF technology is incorporated into weapon detection [37,54], smart homes [121], and healthcare systems. In the future, research will concentrate on creating methods that protect privacy while guaranteeing user confidentiality and data security.
7. Gesture and Sign Language Recognition: When it comes to improving accessibility or recognizing gestures and sign language for device communication, as in [41], RF systems have the potential to be extremely important. Future studies in this area will improve accessibility and human-computer interaction.
8. Micro-Doppler and Sub-Millimeter-Wave RF: The detection of minute human motions and gestures will be made even better by applying sub-millimeter-wave RF and micro-Doppler signatures, as already discussed in [37], opening up new applications in security, healthcare, and other fields.
9. Robustness to Environmental Factors: RF systems must be more resilient to a variety of environmental factors, such as bad weather [59]. The goal of the research will be to create RF systems that perform well in high-stakes environments.
10. Health Monitoring and Aging in Place: RF technology, by tracking daily activities, has the potential to enable aging in place and continuous health monitoring of elderly subjects, as visible in [123,141]. The creation of inconspicuous, long-term monitoring systems that facilitate elderly people’s independent living will be the focus of future directions.

As these future directions unfold, RF sensing for posture and motion detection and classification is poised to have a transformative impact on various industries and improve the quality of life for individuals through innovative healthcare and smart technologies.

## 7. Conclusions

In conclusion, this survey systematically reviewed existing studies on context-aware RF sensing for detection and classification. With a primary focus on the various aspects of RF sensing, we meticulously analyzed the state of the art based on the following criteria: the type of physical sensing used, whether active or passive and examining the input data domains, and distinguishing between time and frequency data. We also explored how spatial diversity in sensing was achieved and the nature of features used, whether raw or statistical. The classification methods implemented, particularly the ML and DL techniques, were evaluated alongside the accuracy and evaluation metrics adopted in each study. Furthermore, our survey delved into the advancements in ML for context-aware sensing, highlighting a variety of use cases and the performance improvements they bring. We also addressed the challenges faced in state-of-the-art RF sensing, such as signal processing issues, data fusion complexities, and the need for real-time responsiveness. Additionally, we identified potential use cases that could benefit from context-aware RF sensing, ranging from healthcare to smart environments. Finally, we discussed future research directions, emphasizing the need for innovative solutions to enhance system robustness, ethical considerations, and the incorporation of emerging technologies. It is our conviction that the way this survey is organized can offer a firm understanding of FL usage in various areas, facilitating the focus on new research directions. 

## Figures and Tables

**Table 1:** Criteria of systematic literature review.

Work	Physical Sensing System [Passive, Active, etc.]	Input Data Domain [Time, Frequency]	Spatial Diversity	Features—What Kind of Data Was Adopted [Raw, Statistic, etc.]	Classification Method	Results (Detection or Classification Accuracy, Error Rate, Latency, etc.)	Activity (Detection, Classification, Application Domain)
[4]	active and passive	NA	NA	NA	YOLO, SSD, Faster-CNN	NA	airborne sensing survey
[33]	passive and active	time domain	multiple radar imagery and ambient EM waves	statistical and raw data	Time Series and Sequence image analysis	NA	airborne sensing
[5]	active—mmWave radar	time and frequency	active and passive mmWave sensors and a depth-sensing camera	raw data	Frequency data analysis	NA	concealed weapon detection
[7]	active	time	analysis of the subjects’ RF reflections	raw data	Bayesian CNN	NA	crowd threat detection
[34]	active radar—SAR	time and frequency	stop-and-go trajectory	raw data	Doppler frequency analysis	3 s data window	through-wall sensing
[35]	active radar	frequency	active sensors	raw data	(single input single output) SISO comms. channel + shallow neural network (SNN)	89.13% accuracy	through-wall sensing
[36]	active radar—UWB	time	active sensors	raw data	CNN	99.7% accuracy	non-contact motion sensing
[37]	active radar	time and frequency	active sensors	raw data—range-Doppler and micro-Doppler signal data	ANN	99.21% accuracy	active shooter and concealed weapon detection
[10]	active—continuous Doppler	frequency	active non-contact sensors	raw data	Frequency data analysis	7.15% error rate	non-contact vital sign detection
[38]	active FMCW radar	frequency	active sensors	raw data	kNN classifier	91.9% accuracy	continuous motion detection and classification
[39]	active radar	frequency	active sensors	raw data—multistatic micro-Doppler data	SVM classifier + diffusion layer	94.72% accuracy	human motion and activity recognition
[40]	active radar—UWB	time and frequency	active sensors	raw data	decision tree, kNN, SVM, Ensemble	94.4% accuracy (in situ motions) and 95.3% accuracy (non-in situ motions)	non-contact motion recognition
[41]	active radar	frequency	active sensors	raw data	decision tree	90% accuracy	gesture recognition
[42]	active—CW radar	time	radio sensors	raw data	CNN	92% accuracy	gesture recognition
[43]	passive radar—ambient IoT devices	frequency	passive IoT sensors	raw data—Doppler spectrograms	Long Short-Term Memory (LSTM) classifier + Visual Geometry Group (VGG) 16 network classifier	91% (occupancy detection) and 91.3% (activity recognition)	occupancy and activity detection
[25]	passive radar—magnetic anomaly detection	anomaly voltage	passive radar sensors	raw data	ANN + kNN-based	95.6% (ANN-based), 98.2% (kNN-based)	passive land mine detection

**Table 2:** Comparison of active and passive RF sensing in literature.

Comparison Criteria	Active RF	Passive RF
Cost	More expensive with regard to the hardware, warranty costs, and failure issues considering that the systems are reliant on locally emitted radio waves [92].	Implementation is reliant on ambient signals such as Wi-Fi, 4G, or 5G signals, and signals from IoT devices, making it comparatively less costly.
Performance	Supports longer range sensing due to control over RF signals emitted. This explains why active radar is popular for long-range air defence [93].	Supports shorter range of sensing due to lack of control over RF signals transmitted [94].
Coverage	Offers more concise target coverage due to greater control of signal properties [95].	Passive RF sensing systems rely on illuminators of opportunity for detection. As a result, their coverage is influenced by the availability and quality of these signals [96].
Flexibility	Offers more flexibility in terms of signal control and customization [95].	Offers less flexibility in terms of signal control and customization [96].
Power Consumption	Higher power consumption due to localized RF wave emission [97].	Lower power consumption due to reliance on externally emitted signals [98].
Interference	Interference-to-Noise Ratio is derived from different types of interference and victim radars and depends on the location of both as well as parameters such as transmit power, antenna gain, and bandwidth. Hence, we have more control over the effects, since we control the source of the signal [99].	More susceptible to direct path interference (DPI), but there are measures, e.g., an adaptive ray to reduce the effect of interference [100].
Regulatory Considerations	Typically require regulatory approval for operation due to their transmission of RF signals. They must adhere to regulations set by government agencies to avoid interference with other RF devices and ensure safety; Operators of active radar systems may need to obtain licences or permits from regulatory authorities to use specific frequency bands or transmit power levels; rely on allocated frequency bands and adherence to spectrum regulations is essential to prevent interference with other users and services.	Due to their reliance on existing signals in the environment, they may have fewer regulatory requirements compared to active radar. Since they do not transmit signals themselves, they may not require specific licenses or approvals for operation; utilize ambient signals such as TV or radio broadcasts, which are already allocated for use by regulatory authorities. As long as they operate within the designated frequency bands and do not cause harmful interference, they may face fewer regulatory hurdles.

## Abbreviations

The following abbreviations are used in this manuscript:

AAL	Ambient Assisted Living
ACM	Active Contour Model
AHI	Advanced Himawari Imager
AI	Artificial Intelligence
ANFIS	Adaptive Neuro-Fuzzy Inference System
ANN	Artificial Neural Network
AUC-ROC	Area Under the Receiver Operating Characteristic Curve
BiLSTM	Bi-directional Long Short-Term Memory
CAMs	Context-Aware Middlewares
CCC	Connected Cruise Control
CG	Control Group
CNNs	Convolutional Neural Networks
CSI	Channel State Information
CWT	Continuous Wavelet Transform
DL	Deep Learning
DRDT	Dynamic Range-Doppler Trajectory
DRL	Deep Reinforcement Learning
DTW	Dynamic Time Warping
EMI	Electromagnetic Interference
EVs	Electric Vehicles
Faster R-CNN	Faster Region Convolutional Neural Network
FFT	Fast Fourier Transform
FG	Fracture (Patients) Group
FMCW	Frequency Modulated Continuous Wave
FoM	Figure-of-Merit
GANs	Generative Adversarial Networks
GPUs	Graphics Processing Units
GRF	Ground Reaction Force
GRU	Gated Recurrent Unit
GVs	Gasoline-Powered Vehicles
HAR	Human Activity Recognition
IoT	Internet of Things
kNN	k-Nearest Neighbor
LSTM	Long Short-Term Memory
MDR	Micro-Doppler Radar
MIMO	Multiple Input, Multiple Output
ML	Machine Learning
mmWave	Millimeter Wave
MODIS	Moderate Resolution Imaging Spectroradiometer
NFN	Neural Fuzzy Network
NsD	Nonsurface Degree
PCA	Principal Component Analysis
PCC	Principal Component Coefficients
PIoTR	Passive IoT Radar
PolSAR	Polarimetric Synthetic Aperture Radar
PID	Polarimetric Intensity Difference
QEA	Quantum-Inspired Evolutionary Algorithm
RBM	Random Body Movement
R-CNN	Region-Based Convolutional Neural Network
RF	Radio Frequency
RL	Reinforcement Learning
RNNs	Recurrent Neural Networks
ROI	Region of Interest
SAR	Synthetic Aperture Radar
SISO	Single Input, Single Output
SNR	Signal-to-Noise Ratio
SSD	Single Shot Detector
SVDD	Support Vector Data Description
SVM	Support Vector Machine
TCNs	Temporal Convolutional Networks
TCR	Target-to-Clutter Ratio
tSNE	t-distributed Stochastic Neighborhood Engineering
UWB	Ultra-Wideband
V2V	Vehicle-to-Vehicle
VAEs	Variational Auto-Encoders
VGG	Visual Geometry Group
WSNs	Wireless Sensory Networks
YOLO	You Only Look Once
